# MINERVA—microbiome network research and visualization atlas: a scalable knowledge graph for mapping microbiome-disease associations

**DOI:** 10.1093/bib/bbaf472

**Published:** 2025-09-23

**Authors:** Saul Langarica, Young-Tak Kim, Adham Alkhadrawi, Jung Bin Kim, Synho Do

**Affiliations:** Department of Electrical Engineering, Pontificia Universidad Católica de Chile, Av. Vicuña Mackenna 4860, Macul, Santiago 7820436, Chile; Department of Radiology, Massachusetts General Hospital and Harvard Medical School, 125 Nashua Street, Boston, MA 02114, United States; Department of Radiology, Massachusetts General Hospital and Harvard Medical School, 125 Nashua Street, Boston, MA 02114, United States; Department of Radiology, Massachusetts General Hospital and Harvard Medical School, 125 Nashua Street, Boston, MA 02114, United States; Department of Neurology, Korea University Anam Hospital, Korea University College of Medicine, 73 Goryeodae-ro, Seongbuk-gu, Seoul 02841, Republic of Korea; Smart Healthcare Research Center, Korea University Anam Hospital, Korea University Medicine, 73 Goryeodae-ro, Seongbuk-gu, Seoul 02841, Republic of Korea; Artificial Intelligence R&D Center, Korea University Anam Hospital, Korea University Medicine, 73 Goryeodae-ro, Seongbuk-gu, Seoul 02841, Republic of Korea; Department of Radiology, Massachusetts General Hospital and Harvard Medical School, 125 Nashua Street, Boston, MA 02114, United States; KU-KIST Graduate School of Converging Science and Technology, Korea University, 145 Anam-ro, Seongbuk-gu, Seoul 02841, Republic of Korea; Kempner Institute, Harvard University, 150 Western Ave, Boston, MA 02134, United States

**Keywords:** microbiome, large language models, knowledge graph, ontology, gut microbes

## Abstract

Bacterial pathogens contribute significantly to the global burden of disease. Understanding their complex interactions with human health is essential for developing new diagnostic, preventative, and therapeutic strategies. While recent breakthroughs have revolutionized our understanding of these relationships, the rapid expansion of microbiome research presents a significant challenge: knowledge remains scattered across scientific literature, hindering comprehensive analysis and clinical translation. To address this, we introduce MINERVA (Microbiome Network Research and Visualization Atlas), an innovative platform that leverages a fine-tuned large language model to systematically map microbe-disease associations across extensive scientific literature. MINERVA constructs a rich, ontology-driven knowledge graph that prioritizes accuracy and transparency, enabling efficient exploration and discovery of previously hidden associations relevant to clinical decision-making. The platform features specialized modules that allow researchers to analyze individual microbes and diseases, visualize complex relationships within the knowledge network, uncover hidden connections through advanced graph algorithms and machine-learning models, and perform personalized and population-level microbiome compositional analysis. These capabilities facilitate the identification of disease risks, comorbidities, and actionable insights, supporting both research and clinical decision-making. By bridging the gap between microbiome research and real-world applications, MINERVA has the potential to transform our understanding of microbe-disease interactions, accelerating discoveries and advancing patient care. The MINERVA platform is available at https://minervabio.org/.

## Introduction

Recent advances and discoveries in biotechnology, DNA sequencing, and new computational tools have revolutionized our ability to study microbial communities in our body, leading to key discoveries about their influence on human health. Under normal conditions, the microbiota serves as a defensive barrier against pathogenic bacterial invasion, thereby bolstering the host’s immune system. Additionally, it aids the host in nutrient absorption and energy derivation from food [1]. On the other hand, an imbalance in the microbiota, termed “dysbiosis,” has been linked to a range of diseases. Research indicates that microbiota plays a crucial role in the onset of cardiovascular diseases, cancer, diabetes mellitus, inflammatory bowel disease, and brain disorders among others [2]. However, this critically important field poses a challenge: knowledge remains scattered across the scientific literature, hindering a comprehensive view of the current state of research and the intricate web of microbiome influences on human health. Moreover, variations in study populations, analytical techniques, and data processing biases can lead to inconsistent results when examining microbial data [3, 4]. Consequently, researchers should not rely on a single source but instead consult a diverse array of resources when investigating microbe-disease connections, making research on the field a daunting task. This underscores the pressing need for sophisticated tools capable of curating and synthesizing the expansive scientific landscape of microbiome research.

Traditional approaches like manual curation of the related scientific literature have led to the development of valuable knowledge bases such as HMDAD [5], gutMDisorder [6], Amadis [7], GMMAD [8], Disbiome [9], and MDIDB [10], among others. These resources provide a strong foundation for researchers, but their reliance on manual curation makes it challenging to keep pace with the rapid growth of microbiome research, which has grown from just a few thousand publications in 2014 to nearly 25 000 publications indexed in PubMed in 2023 alone. Moreover, while these databases excel at the retrieval of curated information, they often lack capabilities to provide advanced insights, such as analyzing interactions among diverse entities or facilitating personalized microbiome analysis tailored to individual contexts.

The advent of natural language processing (NLP) and, more recently, large language models (LLMs) has introduced a transformative paradigm for extracting and synthesizing knowledge from unstructured scientific literature. By processing vast amounts of textual data, these models can automate the identification of microbe-disease associations, offering unprecedented opportunities for scalability and accuracy. However, LLMs are not without limitations. A critical concern lies in their propensity for “hallucination” whereby models generate information not grounded in evidence. This challenge is particularly acute in scientific and clinical domains, where inaccuracies could propagate misinformation with serious implications for human health [11]. For example, studies have documented instances where LLMs generated fabricated citations, misrepresented scientific findings or generated baseless hypothesis [12, 13], leading to the dissemination of misinformation in academic contexts. These issues underscore the need for rigorous validation and transparency when applying LLMs to biomedical tasks.

To tackle these critical challenges, we introduce MINERVA (Microbiome Network Research and Visualization Atlas), an explainable, accurate, and interactive knowledge platform constructed by the automated analysis of a vast corpus of scientific publications on microbe-disease associations using LLMs and an NLP processing pipeline, which maps the extracted knowledge into a knowledge graph. A cornerstone of MINERVA’s design is its commitment to two guiding principles: robustness and explainability. Robustness is ensured through rigorous verification processes while ingesting information, including redundancy strategies that cross-check information both within and between source documents. On the other hand, unlike conventional LLM-based systems, MINERVA grounds all its outputs in verifiable scientific evidence, enabling users to trace each finding back to its source. This combination of robustness and transparency not only minimizes the risks of hallucinations but also empowers researchers to critically assess and trust the underlying data.

**Figure 1:**
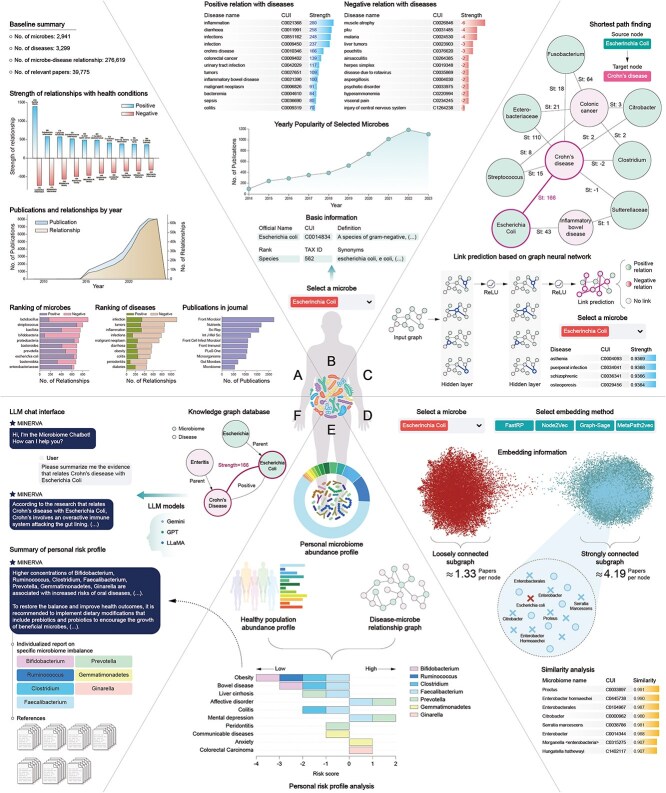
MINERVA provides a suite of modules to explore and analyze microbe–disease relationships, including general statistics of the microbe-disease landscape (A), entity analysis (B), link prediction (C), similarity analysis (D), microbiome risk assessment (E), and an interactive chat interface that integrates a conversational LLM with the knowledge graph for intuitive exploration (F).

MINERVA’s core innovation lies in its ability to transform the fragmented landscape of microbiome research into a structured, interactive, trustworthy, and visually intuitive knowledge graph. Microbes and diseases are represented as nodes, with their relationships, derived from rigorous NLP pipelines, forming the edges. This structured representation is enriched with metadata, evidence links, and relevance scores, creating a robust foundation for advanced analyses. Furthermore, MINERVA provides a suite of interactive tools (Fig. 1) that cater to the diverse needs of microbiome researchers:

- Comprehensive field analysis: Users can explore commonly studied microbes and diseases, identify research trends, and pinpoint understudied areas ripe for exploration.
- Targeted insights: Researchers can delve into specific microbe-disease relationships, accessing detailed evidence and supporting literature.
- Network analytics: Graph-based algorithms can uncover distant associations, clusters, and structural similarities, revealing hidden patterns in the data.
- Predictive modeling: MINERVA’s integration with machine learning algorithms enables tasks such as link prediction and embedding similarity to forecast potential associations.
- Efficient literature synthesis: Leveraging LLM capabilities, the platform generates concise grounded summaries of research findings, highlighting key insights and knowledge gaps.
- Targeted Microbiome Health Assessment: MINERVA enables detailed comparisons between an individual’s or a population’s microbiome profiles and standardized healthy reference datasets. This feature identifies specific microbial imbalances and generates actionable insights by correlating deviations in microbiome abundance to specific health risks.

We envision MINERVA as a valuable resource that will empower microbiome researchers to gain deeper insights, fuel hypothesis generation, and accelerate the development of novel microbiome-based interventions to improve human health.

## Results

### General overview

**Figure 2:**
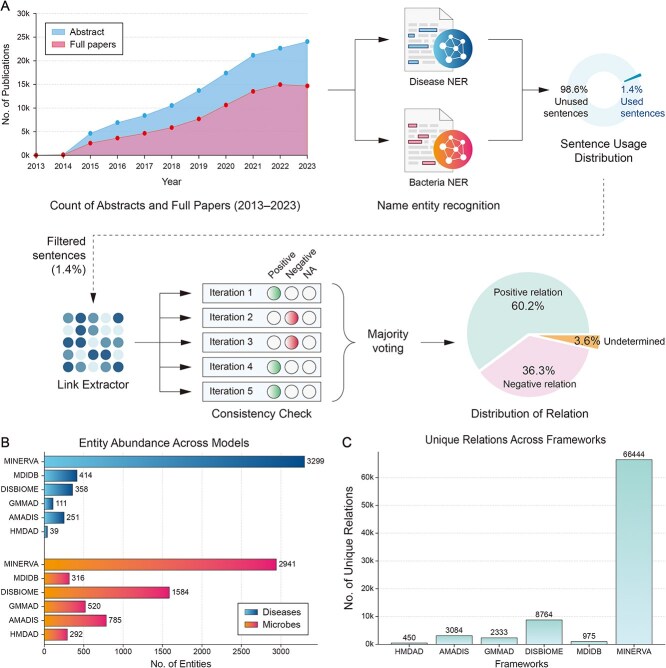
MINERVA has analyzed 129,719 publications with advanced NER and GPT-based LLM models, yielding 66,444 validated microbe–disease relationships across 2,941 microbes and 3,299 diseases, surpassing existing resources.

MINERVA is an automatically constructed knowledge base, which offers a comprehensive and up-to-date understanding of microbiome research that surpasses the scale of manually curated databases. Our automated NLP-based pipeline has processed over 129,719 relevant publications extracted from PubMed (abstracts) or PubMed Central (complete articles when freely available), yielding a wealth of insights that surpasses the scale of any related resource (see Fig. 2). At present, MINERVA houses 3,429 microbes at multiple taxonomic levels, including genus, species and strains, and 35,883 distinct diseases, directly connecting 2,941 microbes with 3,299 diseases through 66,400 distinct relationships. The remaining microbes and diseases not directly connected through explicit microbe-disease relationships are integrated into the knowledge base through hierarchical parent–child associations. This hierarchical structure enables the inference of hidden relationships between microbes and diseases, enriching the knowledge base and facilitating comprehensive exploration of potential connections within the microbiome research landscape.

MINERVA is built upon two fundamental principles: explainability and robustness. Unlike many existing resources that offer limited transparency into the evidence supporting microbe-disease relationships, MINERVA prioritizes explainability through a rigorous extraction process. This process begins with specialized named entity recognition (NER) models that identify relevant microbes and diseases within each sentence of a publication. Only sentences containing both types of entities are then processed by our fine-tuned LLM, which extracts the relationship between them. Using this approach, we are not only able to identify the source publication but also to pinpoint the specific sentences providing the evidence for the extracted relationship. By offering this level of granularity, MINERVA enhances user confidence in the knowledge base and enables deeper exploration of the underlying evidence.

To ensure robustness, we prioritize accuracy over exhaustive coverage by only including in our knowledge base relationships for which our fine-tuned LLM exhibits complete confidence. Furthermore, we employ a redundancy strategy that consolidates evidence from multiple mentions within and across publications, assigning a strength score to each microbe-disease relationship. This multi-layered approach not only reinforces the reliability and trustworthiness of the identified associations but also consolidates current research in case there are contradicting findings between studies. For a comprehensive explanation of these methodologies, including technical details, please refer to the Methods section.

### Modules

MINERVA platform consists of several modules that can be broadly divided into three groups; (i) Exploratory (ii) Knowledge discovery and; (iii) Risk assessment modules. For a detailed tutorial on the use of each module, the reader is referred to Supplementary Material D.

#### Exploratory modules

Among the exploratory modules, the *General Statistics* module (Fig. 1A) provides researchers with a high-level overview of the field, highlighting the most studied microbes and diseases, publishing trends, and key microbe-disease associations. In contrast, the *Individual Analysis* modules (Fig. 1B) offer in-depth information about user-selected microbes (at multiple taxonomic levels) or diseases, including basic information, research trends for that particular entity, its connections within the knowledge graph, and detailed literature evidence supporting these associations. However, for well-studied relationships (e.g. Bacillota with obesity), the volume of supporting evidence can be overwhelming. To address this, MINERVA integrates functionality for generating concise, evidence-based reports using LLMs, grounded in the platform’s database. Supported models currently include GPT-4o, Gemini Pro, and AWS-hosted LLMs, accessible via API keys.

To further enhance exploration and user interaction, the *Chat Interface* module (Fig. 1F) extends the capabilities of MINERVA by leveraging a chat-based LLM grounded on MINERVA’s knowledge. This module allows users to ask any question about the knowledge base, whether it involves exploring specific microbe-disease relationships, retrieving information about individual entities, or even obtaining definitions and broader contextual explanations, ensuring that users are not constrained to predefined queries.

#### Knowledge discovery modules

While MINERVA’s exploratory modules empower researchers to navigate existing knowledge, our platform is uniquely designed to also foster knowledge discovery with a suite of specialized modules.

By harnessing Dijkstra’s [14] shortest-path algorithm, the *Graph Algorithms* module (Fig. 1C) enables the discovery of indirect relationships within the knowledge graph. Users can specify a source and target microbe or disease, and the platform identifies the shortest path, along with alternative routes, connecting these entities. These (possibly) multi-step connections, which may not be immediately apparent, can reveal hidden causal pathways and shed light on complex interactions.

The *Similarity Analysis* module (Fig. 1D) enables researchers to explore the structural relationships within the knowledge graph through a variety of graph embedding algorithms. These include Node2vec [15], FastRP [16], Metapath2vec [17], and Graph Neural Network embeddings [18]. Users can select their desired embedding algorithm, visualize the representation of a chosen microbe or disease in the embedding space, and identify the most similar entities within the same class. Furthermore, the module facilitates the visualization of clusters using the K-means algorithm. Identifying microbes with similar embedding profiles may suggest shared metabolic pathways or ecological niches, while finding diseases clustered together could reveal common etiological factors or potential comorbidities [19].

Finally, the *Link Prediction* module (Fig. 1F) represents a powerful tool for hypothesis generation and knowledge discovery. By leveraging our custom-trained two-layer convolutional Graph Neural Network (see Supplementary Material A for training details), in this module, potential relationships between microbes and diseases that have not yet been explicitly reported in the literature are displayed. Users can select a microbe or disease of interest, and the module will display the most probable positive and negative associations for that particular entity, as predicted by our model.

The current implementation uses Node2vec [15] embeddings, achieving an accuracy and F1-score of ~71% on a held-out test set. The remaining ~30% of misclassifications have important implications: false positives may lead to spurious associations, while false negatives could obscure meaningful ones. As such, predictions from this module should be viewed as hypothesis-generating rather than definitive. We encourage users to validate these findings experimentally or clinically. Future versions of MINERVA will focus on improving performance through richer node features and more advanced graph learning architectures.

#### Risk assessment modules

The risk assessment modules (Fig. 1E and F) represent a key practical application of MINERVA’s knowledge base, providing predictive insights into health risks through microbiome profiling. Users can upload their own or population-level microbial composition profiles and harness MINERVA’s analytical capabilities to explore potential health implications. The platform compares these profiles, at a specified taxonomic level, against a control group, which can be a healthy cohort from GMRepo [20] already integrated into MINERVA or a user-defined dataset. Significant deviations in microbial abundances are automatically flagged and linked to associated diseases within MINERVA’s knowledge graph.

In this context, “risk” refers to a heuristic score that quantifies the cumulative literature-based evidence linking microbial imbalances to specific diseases. Microbial imbalances are defined as taxa whose relative abundances fall outside a configurable reference range (default: 5th–95th percentile) when compared to a healthy population. For each such imbalance, MINERVA queries its knowledge graph to determine whether the imbalance of this specific taxon is positively or negatively associated with any disease. A disease’s risk score is then calculated by summing +1 for each positively associated taxon and −1 for each negatively associated one. This simple yet interpretable scoring system reflects the direction and magnitude of literature-supported associations, offering insights into potential health concerns based on microbiome composition. These scores are intended to inform research and exploratory analyses, not to serve as diagnostic tools.

This streamlined process produces an easily interpretable personalized or population-level risk profile, empowering users to implement proactive health management strategies or advance research into microbiome-associated health risks. Currently, these risk analyses are supported at the genus level; however, support for species-level resolution is planned for a future release to enhance specificity and clinical relevance.

### Comparison with other resources

To assess the robustness and accuracy of MINERVA, we compared it with five well-established, open-source, manually curated resources, as detailed in Table 1. This comparison examined overlaps in covered microbes, diseases, relationships, and their associated labels. To ensure methodological rigor, we aggregated repeated relationships in the benchmark resources using a voting scheme. Beyond direct entity matches, we also considered inferred relationships involving entities one step apart in taxonomic (microbes) or nosological (diseases) hierarchies. This hierarchical approach accommodated variations in granularity across databases, enabling a comprehensive evaluation of MINERVA’s coverage and precision relative to existing resources.

**Table 1:** Comparison of MINERVA with other resources

**Database**	**Overlapping microbes**	**Overlapping diseases**	**Overlapping relationships**	**Differing labels with MINERVA**	**GPT4o coincidence with MINERVA/other DB (%)**	**Gemini’s coincidence with MINERVA/other DB (%)**
AMADIS (*7*)	683 (87%)	206 (82%)	2375 (77%)	1069 (45%)	88.8/3.7	89.2/3.7
GMMAD (*8*)	359 (69%)	108 (97%)	1516 (65%)	636 (42%)	85.3/3.7	85.3/3.7
HMDAD (*5*)	210 (72%)	35 (91%)	274 (61%)	60 (22%)	86.2/6.9	89.7/5.2
DISBIOME (*9*)	1141 (72%)	322 (90%)	5521 (63%)	1932 (35%)	84.5/3.9	84.3/4.3
MDIDB (*10*)	284 (90%)	302 (73%)	897 (92%)	269 (30%)	92.2/3.6	93.4/4.8

Resources are compared in terms of coverage and accuracy. Since there are hundreds of relationships accuracy is assessed using closed-source state-of-the-art LLMs.

From the first and second columns of Table 1, it is evident that MINERVA encompasses most of the microbes and diseases covered in the other databases. However, the percentage of overlapping relationships, as shown in the third column, tends to be lower. This is mostly due to MINERVA’s emphasis on accuracy over exhaustive coverage, as our platform only incorporates relationships where our relation extraction LLM exhibits high confidence.

Notably, among the overlapped relationships, a substantial number of discrepancies were found between the labels assigned by MINERVA and those in other databases, as shown in the fourth column of Table 1. Given the scale of these discrepancies, involving thousands of relationships, we employed OpenAI’s GPT-4o [21] model and Google’s Gemini 1.5 Pro [22] as independent reviewers to assess the conflicting labels. Since the benchmarked resources lack specific evidence for their labels (except for MDIDB [10], which provides one sentence for each relation), we provided some of MINERVA’s compiled evidence as input for the reviewers’ assessment. Remarkably, as demonstrated in the last two columns of Table 1, both closed-source LLMs mostly agreed with MINERVA’s labels in cases of conflict, underscoring the accuracy and robustness of our resource even in comparison to manually curated databases.

**Figure 3:**
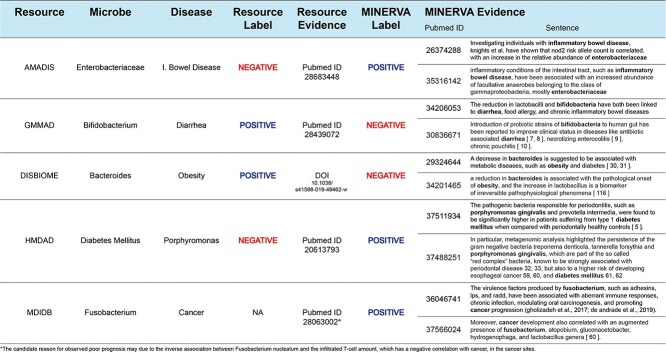
Conflicting labeling and comparison of available evidence between MINERVA and other resources highlight that, unlike most databases which provide little or no supporting text, MINERVA supplies multiple pieces of sentence-level evidence for each microbe–disease relationship, allowing users to thoroughly evaluate the confidence and validity of each label.

This outcome suggests that MINERVA offers a competitive alternative to manually curated databases in capturing nuanced relationships between the microbiome and diseases across a vast body of scientific literature. While manual curation remains valuable, it is often labor-intensive, time-consuming and difficult to scale. In contrast, MINERVA’s transparent, sentence-linked automatically detected associations enable reproducibility, scalability and critical inspection. While we employed LLMs (GPT-4o and Gemini Pro) to independently evaluate conflicting cases, we acknowledge the limitations of relying on models architecturally similar to MINERVA’s own LLM, which may introduce correlated biases. Moreover, discrepancies between MINERVA’s outputs and those of manually curated databases may reflect differences in evidence sources, annotation standards, study sampling, or granularity, rather than differences in objective accuracy alone. As part of future work, we plan to conduct further validation using human expert reviewers to systematically assess and benchmark MINERVA’s outputs against established curated resources. This will be necessary to draw stronger and more definitive conclusions about the system’s accuracy; however, current results are encouraging and suggest that MINERVA is a promising foundation for scalable, literature-grounded discovery. Figure 3 shows some specific examples of MINERVA’s classification labels against other resources. For more examples the reader is referred to Supplementary Material B.

### Case study: population with Alzheimer’s disease

As an example of MINERVA’s ability to effortlessly translate microbiome research into actionable clinical insights, we analyzed a cohort with Alzheimer’s disease presented in [23] available in GMRepo using the *Population Risk Assessment Module*. For this demonstration, the control group from the same study was used as a reference for comparative purposes. Is worth mentioning that the related publication of this study is not among the analyzed publications in MINERVA.

**Figure 4:**
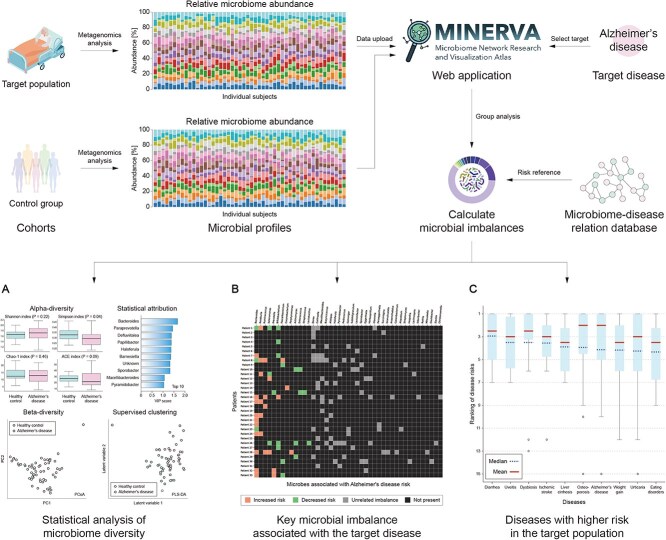
Population risk assessment module. After uploading the microbial profiles of a target population and (optionally) of a control group. MINERVA calculates the microbial imbalances of the target population and delivers multiple insights, which can be broadly divided into three: (A) statistical analysis: Metrics such as $\alpha$-diversity and $\beta$-diversity, along with methods like PLS-DA, are used to compare microbial diversity and identify key discriminative microbes between groups. (B) Disease-specific microbial imbalances: If a target disease is specified, MINERVA will automatically highlight which microbial imbalances are associated to the target disease, providing actionable insights into the microbiome-disease relationship. (C) Risk of diseases: MINERVA calculates and ranks disease risks across the population using its knowledge base, aiding in the identification of potential comorbidities and enhancing diagnostic accuracy. The Population Risk Assessment module analyzes microbial profiles to compare diversity (A), identify disease-specific imbalances (B), and rank disease risks (C), providing actionable insights into microbiome–disease relationships.

As depicted in Fig. 4, after uploading microbiome composition data (see Supplementary Material D.8.1 for additional details on the accepted format) for both the target and control groups, the module delivers three types of results: (i) Statistical Analysis: This includes standard microbiome metrics such as α-diversity (within-sample diversity) and β-diversity (between-group dissimilarity). Additionally, Partial Least Squares-Discriminant Analysis (PLS-DA) [24] is applied to identify microbial genera that best discriminate between the target and control groups based on statistical separation in multidimensional space. This analysis is data-driven and agnostic to disease-specific information, as it does not rely on predefined disease labels or outcomes. (ii) Disease-Specific Microbial Imbalances: This analysis differs from PLS-DA in both purpose and methodology. Instead of identifying statistically discriminative taxa, MINERVA focuses on taxa that are biologically and clinically relevant to a specific disease. Given a selected target condition (e.g. Alzheimer’s disease), MINERVA first retrieves all microbial genera with known associations to the disease based on its literature-derived knowledge graph. It then calculates the abundance of these disease-associated taxa in both the target and control populations, and defines “abnormal” as falling outside a configurable quantile range of the control group distribution (e.g. the 5th to 95th percentile by default). Taxa with such unusually high or low abundances are flagged for further evaluation. Depending on their literature-derived association direction with the disease, they are interpreted as potentially indicative of elevated or reduced disease risk. Unlike purely statistical methods, this approach is grounded in curated biomedical literature and reflects mechanistic or observational evidence, providing disease-specific interpretability, making results more general and actionable in clinical and research settings. In this case, as shown in Fig. 4B, *Bacteroides*, *Roseburia*, and *Ruminococcus* are identified as the most important microbial imbalances associated with Alzheimer’s disease in the analyzed population. (iii) Risks of diseases: Using only the microbial imbalances of each individual, MINERVA is able to compute disease risk scores based on its extensive knowledge graph. These individual risk scores are then aggregated to identify diseases with the highest overall risk. Notably, as shown in Fig. 4C, even without specifying Alzheimer’s disease as the target, it emerged as one of the highest-ranked diseases in the population analysis. This demonstrates MINERVA’s hypothesis-generating capabilities, leveraging microbial data to uncover latent disease associations and providing a powerful tool for population-level risk assessment.

For a more in-depth description and interpretation of the results shown in Fig. 4, including statistical analysis, microbial imbalances, and disease risk rankings, readers are referred to Supplementary Material Section D.9, which provides a detailed walkthrough of the population-level assessments corresponding to this case study. Additionally, section D.8 showcases the usage and outcomes of the *Individual Risk Assessment* module.

## Materials and methods

**Figure 5:**
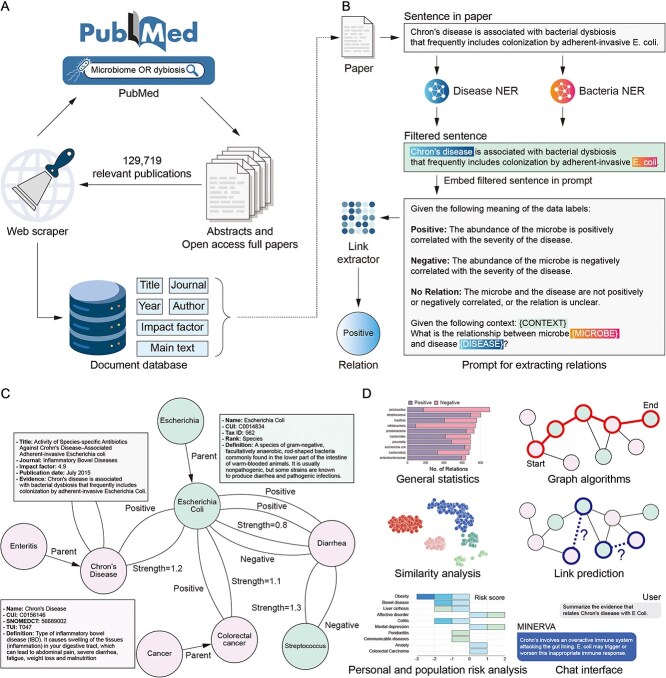
MINERVA’s pipeline (A) collected 129,719 publications from PubMed/PMC, (B) applied NER models and an LLM to extract microbe–disease relationships, (C) validated and integrated these relationships as triplets into a knowledge graph, and (D) developed a user-friendly platform with analytical modules for exploration and discovery.

The construction of MINERVA followed a systematic four-step pipeline (Fig. 5): (i) Data collection from PubMed and PubMed Central, (ii) Data processing using specialized NER models and LLMs to extract meaningful microbe-disease relationships, (iii) Construction of a knowledge graph, and (iv) Development of an intuitive web-based platform to enable seamless exploration, analysis, and hypothesis generation.

### Data collection

To populate MINERVA, we collected 129,719 relevant PubMed abstracts published between 2014 and 2023, obtained by the search query *((microbiome) OR (dysbiosis) OR (microbiome alterations)) AND (“2013”[Date - Create]: “023”[Date - Create])* (see Fig. 5A). For those abstracts where the full paper was freely available on PubMed Central (PMC), we obtained the full paper as well, resulting in 78 382 full papers.

As depicted in Fig. 2, the number of published microbiome studies has grown substantially in recent years. This rapid expansion of research in this field underscores the need for automated solutions like MINERVA, empowering researchers to efficiently navigate and leverage the latest discoveries in this dynamic area.

**Table 2:** Comparison of different open-source LLMs for relation extraction

**Model**	**Accuracy**	**F1-score**	**Accuracy (F.C.)**	**F1-score (F.C.)**	**Coverage (F.C.)**
PubMed-BERT (Finetuned) [38]	0.790	0.777	-	-	-
BioLink-BERT (Finetuned) [39]^a^	0.797	0.787	-	-	-
Mixtral 8 × 7b (Zer-Shot) [37]	0.610	0.636	0.695	0.716	59%
Mixtral 8 × 7b (Few-Shot)	0.801	0.782	0.832	0.812	91%
Llama 2 70b (Zero-Shot) [40]	0.217	0.110	0.213	0.08	66%
Llama 2 70b (Few-Shot)	0.765	0.741	0.805	0.778	86%
Orca 13b (Zero-Shot) [41]	0.658	0.603	0.715	0.655	73%
Orca 13b (Few-Shot)	0.673	0.634	0.751	0.713	68%
Mistral 7b (Finetuned) [42]	0.818	0.806	0.907	0.893	66%
Zephyr 7b (Finetuned) [43]	0.828	0.814	0.920	0.902	65%
BioMistral 7b (Finetuned) [36]	0.824	0.808	0.881	0.859	78%
**BioMistral-AUG-7b (Finetuned)**	0.847	0.841	0.890	0.884	81%

^a^Best performing model in (*29*).

The comparison is done using a five-fold validation approach on (*29*) dataset. The objective is to find the best possible trade-off between accuracy and coverage of full-confidence (F.C.) prediction.

### Data processing

For each relevant publication (abstract or full paper), we split it into sentences and analyzed each sentence independently using two NER models, one trained to identify microbes and the other trained to identify diseases. If a sentence contains at least one microbe and at least one disease, we used our fine-tuned LLM to infer the relationship between the microbe(s) and disease(s) present in the sentence (Fig. 5B). Sentences lacking either entity were excluded from further analysis.

#### Name entity recognition

Unlike previous studies that have used dictionary-matching techniques for disease and microbe entity recognition from scientific publications [10, 25], in this work we adopted transformers-based NER models based on the BERT architecture [26], due to its superior performance and generalization [27].

Specifically, we used the SciBERT model [28] for disease NER, and a custom microbial NER solution by finetuning the DistilBERT model for token level classification [29] on the BNER2.0 dataset [30], which consists in more than 12 480 labeled microbial entities across 23 174 sentences. Training was performed using cross-entropy loss, with a batch size of 32, a learning rate of 2e-5, and early stopping based on validation loss to prevent overfitting. To ensure consistency and reproducibility, we followed the same dataset splits (training, validation, and test) provided by the original BNER2.0 authors and available through their public GitHub repository provided in [30]. Using this model we achieved, an F1 score of 0.914, a precision of 0.951, and a recall of 0.895 over the test set which consists in 2043 bacterial entities across 3053 sentences. Following entity identification, we employed ScispaCy’s Entity Linker [31] for normalization, assigning a UMLS Concept Unique Identifier [32] to each recognized entity to ensure standardized representation and facilitate downstream analysis.

#### Relation extraction

Relation extraction was performed using a fine-tuned GPT-based LLM, specifically using its ability to understand nuanced scientific language and complex relationships within the text [33]. We fine-tuned and evaluated the model using the dataset provided by [34], a collection of 1,100 manually labeled sentences categorizing microbe-disease relationships as Positive (microbe promotes the disease), Negative (microbe inhibits the disease), Related (unclear direction of the relation), or NA (unrelated). To improve clarity and focus on the directionality of the relationships, which is an important feature of MINERVA, we merged the Related and NA labels into a single Unrelated category. This resulted in a refined dataset with 571 Positive, 305 Negative, and 224 Unrelated sentences.

Using this dataset, we compared the performance of several open-source fine-tuned and non-fine-tuned models under zero-shot and few-shot conditions, employing a five-fold validation approach. Since [34] found BERT-based models to perform competitively with or even surpass GPT-based models on this task, we included them in our evaluation. Fine-tuning of LLMs was carried out using conventional supervised fine-tuning using a prompt-based approach. Closed-source LLMs were excluded due to the prohibitive cost of applying them to large-scale scientific literature analysis.

In the case of GPT-based models, we applied self-consistency [35], which usually consists of sampling multiple answers with varying temperature values (a parameter that controls the randomness of the model’s output) and prioritizing answers achieving a majority vote. While the majority approach is useful in general multiple-choice tasks, for our high-confidence knowledge graph construction, we just included relations with complete consistency. The remaining relations were discarded, prioritizing accuracy over wide coverage.

Table 2 reveals that few-shot learning significantly improves non-finetuned models. However, fine-tuning, even on smaller models, consistently outperforms non-finetuned approaches. This is likely due to the highly technical scientific terminology prevalent in microbiome-related publications, which fine-tuning helps the models to better understand and process. Among the finetuned models, the Biomistral 7b model [36] achieved the best trade-off between accuracy and coverage for full-confidence (F.C.) predictions. We then proceeded to boost its performance even further by using a data-augmentation approach. This approach leveraged the Mixtral 8 × 7b LLM [37], prompting it to generate two rephrased version of each sentence in our database that described the relationship between a given microbe and disease. Then for these rephrased sentences, we replaced the original microbe and disease by randomly chosen entities from the original database. While this strategy could introduce biologically implausible combinations, we found that, in practice, it improved generalization and performance. We used these additional sentences to augment the database and train the model with them. Training on this augmented dataset yielded Biomistral-AUG 7b, which demonstrates the best overall performance and the best trade-off between accuracy and coverage for F.C. predictions. The specific prompt used for the zero-shot, few-shot, and fine-tuning approaches is presented in the Supplementary Material C.

### Knowledge graph construction

**Figure 6:**
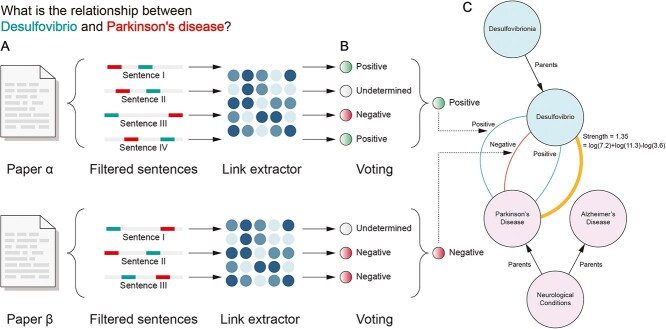
MINERVA’s knowledge graph is built through a three-step process that requires full LLM confidence (A), applies majority voting within publications (B), and updates relationship strength across studies to ensure accuracy and reliability (C).

In addition to only considering the high confidence relations predicted by our LLM (Fig. 6A), to further ensure the precision of our knowledge graph, we employed a two-tiered approach. First, we addressed potential discrepancies within a single paper, that is, for a microbe-disease pair discussed in multiple sentences within the same paper, if those sentences presented conflicting relationship labels (Positive, Negative, or Unrelated), a majority voting approach determined the final label (Fig. 6B). Prioritizing clarity and directionality, we retained only Positive and Negative relationships, discarding Unrelated ones. This ensures our knowledge graph captures only strongly verified associations, which are then incorporated as triplets ($m,r,d$). Here, $m$ and $d$ represent microbe and disease nodes respectively, and $r$ is the relationship between them, represented as an edge in the knowledge graph.

Secondly, to resolve potential conflicts in microbe-disease relationships across multiple papers, we implemented a weighting approach, that consists of the following: Given $N$ relationships extracted from different papers between microbe $m$ and disease $d$, we calculate the strength of the association between them as follows:


(1) \begin{equation*} {w}_{md}={\sum}_{k=1}^N{r}_{k_{md p}} \end{equation*}

With ${r}_{k_{mdp}}$ as the strength of the relationship $k$ between $m$ and $d$ coming from paper $p$, which is given by:


(2) \begin{equation*} {r}_{k_{mdp}}=\mathit{\operatorname{sign}}\cdot lo{g}_{10}\left({F}_p\right) \end{equation*}

Where ${F}_p$ is the impact factor of the journal in which paper $p$was published and $\mathit{\operatorname{sign}}$ is +1 if the relationship is labeled as positive and −1 if the relationship is labeled as negative. We slightly favor papers published in more prestigious journals, as these publications generally undergo a more rigorous peer-review process, potentially increasing the reliability of their findings (Fig. 6C).

Finally, we enriched the knowledge graph by incorporating hierarchical relationships. Disease entities were expanded using the SNOMED CT ontology [44], while taxonomical structures were applied to microbes, adding parent and child nodes. This enrichment broadens the graph’s scope and enhances its utility for analyzing multi-hop microbe-disease connections across hierarchical trees.

### MINERVA’s interface

Finally, for the practical implementation of the knowledge graph we used Neo4j [45] and to create MINERVA’s user-friendly interface, we employed the Streamlit framework [46], which allows for the rapid development of interactive, data-driven web applications. MINERVA’s platform is freely available at https://minervabio.org/, and a video showcasing its use is provided in Supplementary Video S1.

## Discussion

MINERVA represents a significant advancement in navigating the complex and rapidly expanding landscape of microbiome research. It provides a comprehensive, explainable, and up-to-date platform designed to systematically map and analyze known and potential microbiome-disease associations derived from a vast corpus of scientific literature. By integrating advanced NLP techniques, rigorous validation strategies, graph algorithms, and machine learning models, MINERVA offers an intuitive and interactive interface. This empowers researchers to efficiently conduct systematic explorations, uncover hidden patterns within extensive datasets, and generate novel, data-driven hypotheses, thereby accelerating the pace of discovery in this vital field.

Although the field has long benefited from manually curated databases such as Disbiome [9], Amadis [7], gutMDisorder [6], HMDAD [5], and GMMAD [8], as well as more recent efforts like BugSigDB [47], BGMDB [48] and MIMIKG [49], their reliance on manual effort limits scalability and responsiveness to the rapid growth in microbiome literature, often resulting in delays in incorporating new findings. Moreover, while effective for retrieving established facts, these databases typically lack integrated analytical tools for advanced network-based exploration, hypothesis generation, or predictive modeling.

To address the limitations of manual curation, early automated approaches based on traditional NLP techniques have been developed. These systems often employed dictionary-based NER and rule-based or co-occurrence methods to extract relationships, examples include PubTator [50], SemMedDB [51], and Alvis-based [52] pipelines. While these methods offer improved scalability, they struggle with the linguistic complexity of scientific literature, frequently misinterpreting speculative, negated, or non-assertive statements [53]. As a result, they tend to lack the precision and contextual depth needed to accurately capture the nature and direction of microbe-disease relationships.

Recent efforts, such as GDReBase [54] and MarkerGenie [55], have leveraged deep learning and transformer-based models to improve relationship extraction. However, many of these systems still fall short in transparency and explainability. Often, users cannot trace the output back to specific pieces of textual evidence, making it difficult to assess the reliability of the extracted relationships or validate findings independently. This gap between automation and interpretability remains a key limitation in current AI-based solutions.

MINERVA builds upon these advancements while introducing several key innovations that address the limitations of prior approaches. First, it leverages a state-of-the-art, fine-tuned LLM specifically optimized for extracting directional microbe-disease relationships, capturing nuanced associations often missed by traditional NLP or earlier ML models. Second, MINERVA places a paramount emphasis on explainability and robustness. Unlike systems where the link between data and source can be opaque, MINERVA grounds *every* extracted relationship in specific source sentences within publications. Its multi-tiered validation process, requiring high LLM confidence, employing within-paper majority voting, and using a publication-weighted aggregation across papers, is explicitly designed to ensure high accuracy and minimize the risk of LLM “hallucinations, a critical concern in scientific domains. This transparency allows users to critically assess the evidence underpinning each association.

Third, MINERVA extends beyond static knowledge representation by integrating a suite of analytical tools for exploration and discovery. These include modules for graph traversal (Shortest Path), structural embeddings (Similarity Analysis), machine learning–based link prediction (Graph Neural Networks), and microbiome-based risk assessment (Individual and Population Risk Modules). Notably, the link prediction model achieved an F1-score of 73.1% on a held-out test set using Node2vec embeddings (Table S1), while the relation extraction model reached an F1-score of 88.4% on F.C. predictions (Table 2). This integration of a high-fidelity knowledge graph with advanced analytical and predictive capabilities distinguishes MINERVA from many existing resources that primarily focus on data retrieval.

For instance, as highlighted in the case study of the population with Alzheimer’s disease (Fig. 4), MINERVA enabled the rapid identification of relevant microbial imbalances and associated disease risks directly from uploaded microbiome data, linking statistical findings to the vast underlying knowledge graph, a capability extending beyond simple literature search or database lookup. In clinical practice, such insights could guide physicians in modulating an individual’s microbiome to counteract patterns associated with disease, fostering personalized medicine approaches through targeted interventions. This underscores MINERVA’s potential to bridge the gap between raw scientific data and actionable clinical insights, serving as a catalyst for translating microbiome research into tangible advancements in patient care.

### Limitations

Despite these strengths, users should remain aware of important limitations. The platform currently relies on open-access abstracts and full-text articles from PubMed and PubMed Central, which may introduce selection bias by excluding content from paywalled or non-indexed journals. In addition, our pipeline focuses on extracting relationships at the sentence level. This may overlook more complex associations that span multiple sentences or require co-reference resolution and discourse-level analysis, an area we plan to address in future iterations.

The weighting mechanism, which prioritizes information from high-impact journals, does not currently account for other study quality indicators such as sample size, replication, or methodological rigor. Furthermore, MINERVA prioritizes precision over recall by including only relationships where the model exhibits F.C.. While this reduces false positives, it may exclude context-dependent or low-frequency but biologically meaningful associations. False positives and false negatives have different but equally important implications. A false positive (i.e. a spurious microbe–disease link) could lead researchers to investigate invalid hypotheses, potentially misallocating resources. Conversely, a false negative might obscure relevant associations that could contribute to understanding disease etiology. Therefore, is important to emphasize that all insights generated by MINERVA, including predicted associations and risk assessments, are inferred from published literature and computational models and should be interpreted as hypothesis-generating, not diagnostic. Independent experimental or clinical validation should be done before being applied in real-world diagnostic or therapeutic contexts.

### Future work

To address these limitations and advance MINERVA, we will pursue several strategic directions. First, we aim to expand the knowledge base by incorporating additional repositories beyond PubMed, including clinical trial databases, longitudinal metagenomics datasets, and metabolomics resources. This will reduce bias and foster a more holistic understanding of microbiome compositions and their health implications [56]. In parallel, we plan to implement an automated update mechanism that regularly queries PubMed and PubMed Central using our original keyword strategy, filtering by publication date and excluding already processed entries. New articles will be integrated via the existing NLP pipeline, allowing MINERVA to stay continuously up-to-date and relevant [57]. We also plan to enhance the NLP pipeline by implementing cross-sentence relationship extraction and coreference resolution to capture more complex associations, similar to the work in [58]. Additionally, we will develop a refined weighting strategy incorporating factors like study size, methodological rigor, and replication status, moving beyond journal impact factors alone. These improvements will transform MINERVA into a more powerful tool, generating nuanced insights while maintaining accuracy and transparency.

Another promising future direction is the integration of additional entities into the knowledge graph, such as diet, nutrition, environmental exposures, lifestyle factors, and pharmaceutical interventions impacting the microbiome [59]. By broadening MINERVA’s scope, the platform could provide a more holistic understanding of the complex interplay between the microbiome, host factors, and human health.

In conclusion, MINERVA represents a step forward in microbiome research, hypothesis-generation, and clinical application. This comprehensive, explainable, and interactive platform leverages an extensive database and cutting-edge computational tools to systematically map and analyze microbiome-disease associations within a knowledge graph. By providing researchers and clinicians with streamlined access to microbiome patterns linked to specific diseases and offering tools for hypothesis generation and prediction, MINERVA facilitates the identification of actionable insights, potentially enabling interventions to prevent comorbidities or mitigate disease progression. While challenges remain, MINERVA provides a valuable tool for advancing our understanding of the microbiome’s role in human health. With further refinement and broader integration, MINERVA is poised to become an indispensable resource for both clinical applications and microbiome research, contributing to progress in precision medicine and public health.

**Key Points**

- **Addressing Scattered Knowledge:** Microbiome research is rapidly expanding, but scattered knowledge across scientific literature hinders comprehensive understanding and clinical application.
- **AI-Powered Knowledge Graph:** Microbiome Network Research and Visualization Atlas (MINERVA) uses advanced AI techniques to create a scalable knowledge graph that maps microbe-disease associations from over 129,000 publications.
- **Ensuring Accuracy and Transparency:** The platform prioritizes accuracy through rigorous verification processes and allows users to trace information back to original sources.
- **Tool for Research and Clinical Insights:** MINERVA offers tools for exploring trends, discovering hidden relationships, and predicting new associations, with the potential to accelerate research and inform clinical decision-making.

## Supplementary Material

Def_Supplementary_Material_bbaf472

MINERVA_tutorial_bbaf472

## Data Availability

All data, and code for reproducibility is available at https://github.com/MGH-LMIC/MINERVA. MINERVA’s platform is freely available at https://minervabio.org/. Additionally, all the entities and found relationships are available as a supplementary material.
