# Supplementary material for: MINERVA—microbiome network research and visualization atlas: a scalable knowledge graph for mapping microbiome-disease associations
Source: Brief Bioinform. 2025 Sep 23;26(5):bbaf472. doi: 10.1093/bib/bbaf472 (PMC12454267; doi:10.1093/bib/bbaf472)
Supplement: Def_Supplementary_Material_bbaf472 [file def_supplementary_material_bbaf472.docx]

**Supplementary Material:**

**MINERVA – Microbiome Network Research and Visualization Atlas:**

A Scalable Knowledge Graph for Mapping Microbiome-Disease Associations

**Authors:** Saul Langarica^1,2^, Young-Tak Kim^1^, Adham Alkhadrawi^1^, Jung Bin Kim^3^, Synho Do^1,4,5*^

**Affiliations:**

^1^ Laboratory of Medical Imaging and Computation, Department of Radiology, Massachusetts General Hospital and Harvard Medical School, 125 Nashua Street, Boston, Massachusetts, USA.

^2^ Department of Electrical Engineering, Pontificia Universidad Católica de Chile, Av. Vicuña Mackenna 4860, Macul, Santiago, Chile.

^3^ Department of Neurology, Korea University Anam Hospital, Korea University College of Medicine, 73, Goryeodae-ro, Seongbuk-gu, Seoul, 02841, Republic of Korea.

^4^ KU-KIST Graduate School of Converging Science and Technology, Korea University, 145, Anam-ro, Seongbuk-gu, Seoul, 02841, Republic of Korea.

^5^ Kempner Institute, Harvard University, 150 Western Ave, Boston, Massachusetts, USA.

*Corresponding author.

**A. LINK PREDICTION MODULE**

To develop the *Link prediction* module, we implemented a graph neural network (GNN) model capable of predicting potential relationships between microbes and diseases not explicitly stated in the literature. We implemented a two-layer convolutional GNN architecture with ReLU activation functions as our graph encoder and a two-layer feed-forward neural network as the decoder, using PyTorch Geometric library [1].

The link prediction model was trained to classify potential links into three categories: No Link, Positive Relation, or Negative Relation. To optimize the model's performance, we experimented with different types of node features given as input to the model: (i) No Features: The GNN operated solely on the graph's structure, without any additional node information. (ii) Entity Definition Embeddings: We used a pre-trained sentence transformer [2] to generate embeddings of the textual definitions of each microbe and disease entity. (iii) Node2vec Embeddings: We employed Node2vec [3] to learn low-dimensional representations of nodes based on their structural roles in the graph. And (iv) Metapath2vec Embeddings: A similar technique to Node2vec but designed for heterogenous graphs [4].

To ensure a robust evaluation, we constructed our test set comprising 6,617 relationships where MINERVA's labels aligned with those of the benchmarked manually curated databases (see columns 3 and 4 in Table 1 in the main text). The remaining relationships were randomly split into training (80%) and validation sets (20%).

Table S1 presents the performance of the GNN on the test set, showcasing the results for each type of node embedding. Notably, both Node2vec and Metapath2vec embeddings, which capture the structural context of nodes within the graph, lead to the highest accuracy and F1 scores. However, the model without node embeddings follows closely, suggesting that the graph structure itself contains valuable information for link prediction, and node embeddings just offer a marginal performance improvement.

We ultimately selected the Node2vec embedding-based model as our final link prediction model due to its superior overall performance across the evaluated metrics. Future work will explore more sophisticated GNN architectures and the integration of additional node features, such as genomic data for microbes or disease ontology information, to further enhance link prediction accuracy.

| **Model** | **Accuracy** | **Precision** | **Recall** | **F1-Score** |
| --- | --- | --- | --- | --- |
| Metapath2vec | 0.709 | 0.791 | 0.706 | 0.729 |
| Node2vec | 0.713 | 0.780 | 0.713 | 0.731 |
| Sentence Embedding | 0.660 | 0.660 | 0.808 | 0.696 |
| No Embedding | 0.702 | 0.778 | 0.699 | 0.725 |

**Table S1: Link Prediction results for different types of node embeddings**

**B. ADDITIONAL EXAMPLES OF MINERVA EVIDENCE VS OTHER RESOURCES**

| **Resource** | **Microbe** | **Disease** | **Resource Label** | **Resource Evidence** | **MINERVA Label** | **MINERVA Evidence** |
| --- | --- | --- | --- | --- | --- | --- |
| AMADIS | Bifidobacterium | Obesity | POSITIVE | PMID:  32309947 | NEGATIVE | - **27845741**: Furthermore, an increase in the population of bifidobacterium exerts anti-obesity and lipid-lowering effects against high fat.  - **35592636**: The use of probiotics containing lactobacillus and bifidobacterium species in obesity treatment is promising. |
| AMADIS | Escherichia Coli | Immflamation | NEGATIVE | PMID:  30897686 | POSITIVE | - **37685055**: escherichia coli infection can disrupt this balance, increasing the abundance of gram-negative bacteria, increasing inflammation and oxidative damage, and disrupting the barrier function.  - **34938203**: Interestingly, c. tropicalis was shown to positively correlate with serratia marcescens and escherichia coli in cd, further supporting their role in sustaining chronic inflammation as a “team” in the commensal niche (hoarau et al., 2016). |
| GMMAD | Lactobacillus | I. Bowel Disease | POSITIVE | PMID:  28039159 | NEGATIVE | - **35215426**: Rosen et al. (2017) reported that inflammatory bowel disease was related to decreased abundance of microbes with anti inflammatory potential (such as bifidobacterium and lactobacillus).  - **34641619**: In another trial, after patients with inflammatory bowel disease (inflammatory bowel disease) consumed yogurt, probiotics such as bifidobacterium and lactobacillus in the patients’ intestines increased, which helped improve intestinal function |
| GMMAD | Prevotella | Arthritis Rheumatoid | NEGATIVE | PMID:  18528968 | POSITIVE | - **30510245**: Thus, prevotella species are primary suspects also in humans, in which the increased abundance of these bacteria at mucosal sites has been associated with th17 mediated diseases including periodontitis 24 and rheumatoid arthritis 48  - **36933668**: In fact, numerous studies have identified an association between a disproportionate abundance of members of the prevotella taxa and a range of infections and inflammatory conditions including rheumatoid arthritis, intestinal and vaginal dysbiosis, metabolic disorders and major depressive disorder. |
| HMDAD | Collinsella aerofaciens | Colon Cancer | POSITIVE | PMID:  7574628 | NEGATIVE | - **33615992**: Moreover, collinsella aerofaciens has been associated with a low risk of colon cancer, and patients with ibd show lower gut levels of this genus than do control individuals.  - **32882999**: Collinsella aerofaciens has been associated with a low risk of colon cancer and ibs. |
| HMDAD | Bilophila | Liver cirrhosis | NEGATIVE | PMID:  25079328 | POSITIVE | - **36957974**: Patients with cirrhosis showed higher abundance of enterococcaceae, gemellaceae at family level and phascolarctobacterium, enterococcus, streptococcus, gemella and bilophila at genus level in patients with hcc  - **35897739**: Several in depth studies of patients with hcc have established associations between particular microbiome compositions and the development of hcc. For example, genera, such as bacteroides, phascolarctobacterium, enterococcus, streptococcus, gemella, bilophila, are increased in patients with hcc from nafld and cirrhosis compared to healthy controls. |
| DISBIOME | Streptococcus | Pneumonia | NEGATIVE | DOI:10.1017- S00071145 - 19001909 | POSITIVE | - **35036248**: There are some studies that have reported the increased abundances of lachnospiracea e and ruminococcaceae in subjects with an insulin resistant status, such as diabetic disease and obesity.  - **37891977**: While grape pomace promotes the decrease of enterobacteriacae and escherichia coli [ 251 ], a combination of quercetin and resveratrol leads to a reduced relative abundance of desulfovibrionaceae, acidaminococcaceae, coriobacteriaceae, bilophila, and lachnospiraceae (all possibly linked to diet induced obesity). |
| DISBIOME | Lactobacillus | Hypertension | POSITIVE | DOI:10.3389-fphar.2020 - 00258 | NEGATIVE | - **36674891**: Lactobacillus is also able to help ameliorate hypertension by secreting substances working as th 17 lymphocytes inhibitors that decrease inflammation  - **33212807**: The authors observed significantly lower association between the presence of lactobacillus in the group of patients with diabetes and hypertension, and higher in the diabetes only, and diabetes with hyperlipidemia cohorts. |
| MDIDB | Bifidobacterium | Melanoma | RELATE | PMID^^[[1]](#footnote-1)^^:  31555274 | NEGATIVE | - **29872574**: Matson et al. 10 collected 38 stool samples from melanoma patients on anti pd 1 treatment and after 16s RNA sequencing and quantitative PCR analysis he identified bifidobacterium spp, lactobacillus animalis, roseburia intestinalis and veillonella parvula as bacteria associated with beneficial response.  - **33062956**: The presence of bifidobacterium in combination with anti pd l1 treatment can result in almost complete inhibition of melanoma tumor growth. |
| MDIDB | Helicobacter pylori | Ulcer | NEGATIVE | PMID^^[[2]](#footnote-2)^^:  29576949 | POSITIVE | - **34880265**: Perhaps the best known association is of bacteria (helicobacter pylori) causing gastric ulcers that progress into gastric cancer  - **37600949**: helicobacter pylori (helicobacter pylori) is a bacterium that can live in the stomach and has been linked to many digestive disorders, including gastritis, stomach ulcers and stomach cancer. |
| MDIDB | Bacteroides fragilis | inflammatory bowel disease | NEGATIVE | PMID^^[[3]](#footnote-3)^^:  31448244 | POSITIVE | - **35985169:** these include enterotoxigenic bacteroides fragilis, which produces b. fragilis toxin (b. fragilis toxin) that has been associated with inflammatory bowel disease, acute diarrhea, and colorectal cancer  -**36943061:** bacteroides fragilis is among the most abundant and pathogenic bacterial species in the gut microbiota and is associated with diarrheal disease in children, inflammatory bowel disease, and the development of colorectal cancer |
| MDIDB | Escherichia | Colitis | NEGATIVE | PMID^^[[4]](#footnote-4)^^:  27770517 | POSITIVE | -**34444821:** a high fat, high carbohydrates and high sugar diet leads to an increase in prevotella, bacteroides and escherichia, which are involved in colitis  -**35762770:** escherichia was higher in colitis induced mice than in healthy ones and thus probably linked to uc incidence |
| MDIDB | Parabacteroides distasonis | Colitis | POSITIVE | PMID^^[[5]](#footnote-5)^^:  26727498 | NEGATIVE | -**37174660:** this allowed us to characterize bacteria with high potential in the context of ibd, notably three parabacteroides distasonis strains able to counteract colitis in mice  -**36726819:**reversely, in recent studies, oral administration of parabacteroides distasonis antigen was found to reduce experimental murine colitis by modulating immune and microbiota composition (71), which may need to be further investigated. |

**Table S2: Additional examples of discrepancies between MINERVA and other resources**

**C. PROMPT FOR RELATION EXTRACTION**

**
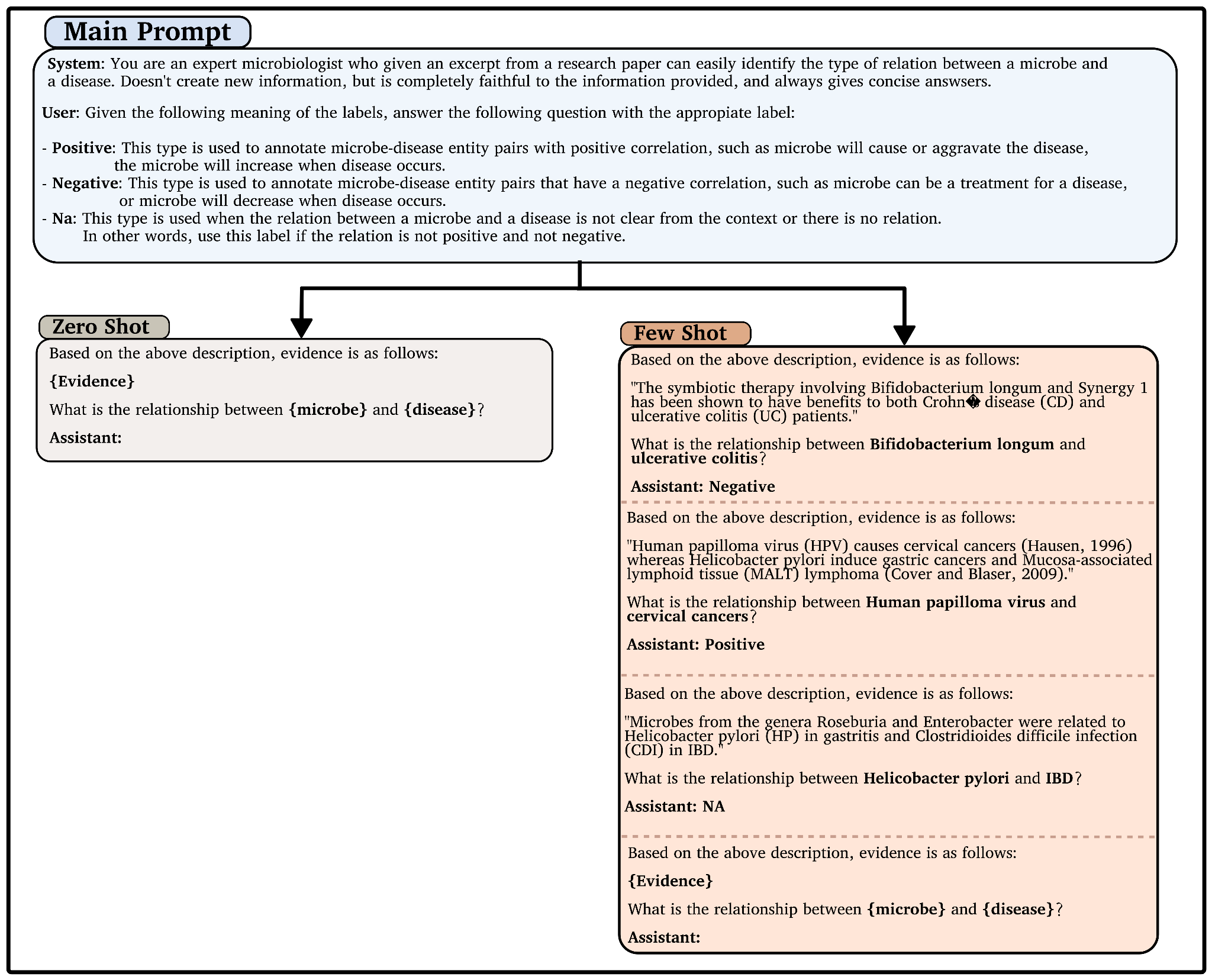
**

**Figure S1: Prompt for relation-extraction models. Prompt templates for zero-shot (left) and few-shot (right) models. Fine-tuned models utilized the zero-shot prompt template.**

**D. FULL MINERVA USAGE TUTORIAL**

In the following, a step-by-step guide to using the MINERVA platform, accessible at<https://minervabio.org> will be described.

**D.1 LOGIN PAGE**

The first page presented is the login screen. Users can sign in using MINERVA-specific credentials, which can be created via the sign-up page, or alternatively, log in with their Google account. After logging in, users may provide their OpenAI, Gemini, or AWS deployed-models API keys to enable the platform's large language model. capabilities.

**
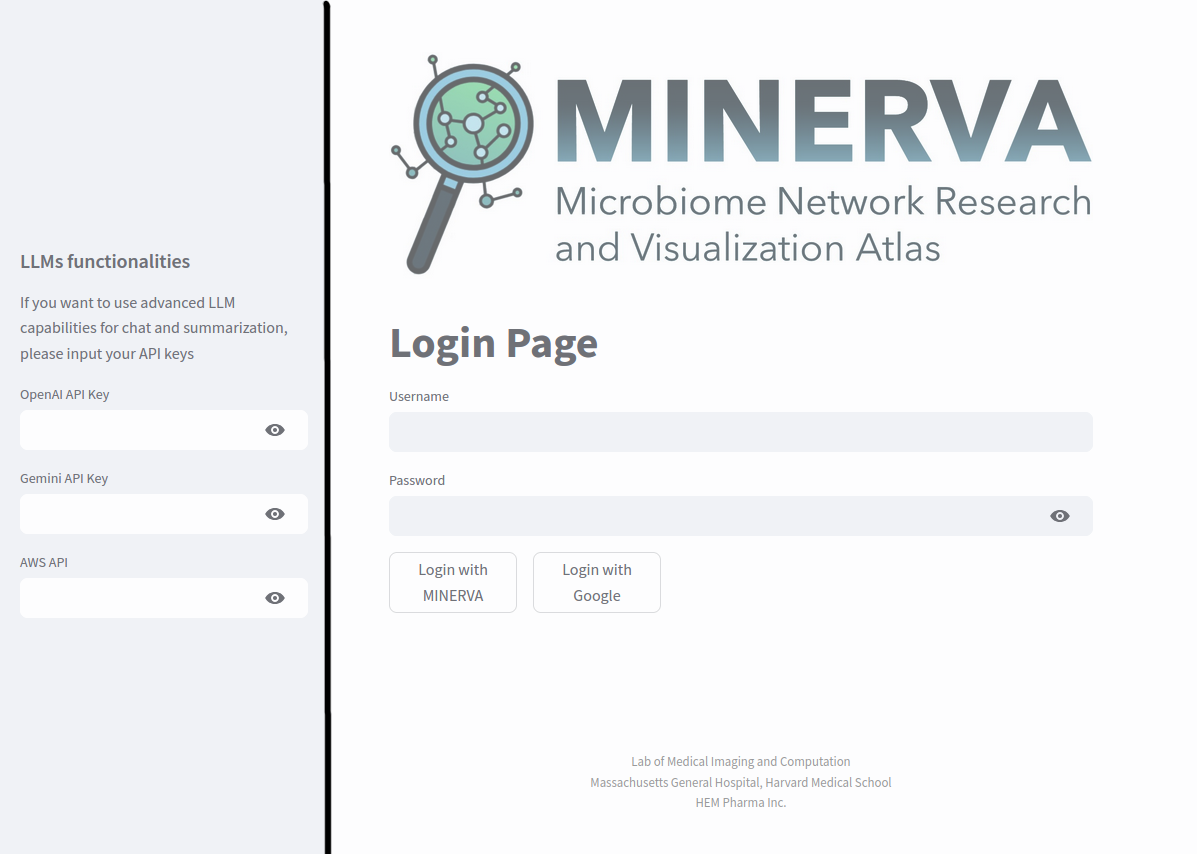
**

**Figure S2: Login Page and LLM API key**

**D.2 GENERAL STATISTICS**

The *General Statistics* module provides a high-level overview of the contents and scope of MINERVA’s knowledge base. It highlights the most frequently studied microbes and their associated diseases, based on the number of extracted relationships and supporting publications. Additionally, it displays the journals that have contributed the most relevant publications to the database, offering insight into key sources driving microbiome research. As shown in the bottom right corner of Figure S3, the number of microbiome-related publications has grown steadily over time. The module also includes rankings of the most positive and most negative microbe–disease relationships in the knowledge base, based on the volume of supporting evidence extracted from the literature. This comprehensive snapshot allows users to quickly grasp research trends, frequently studied entities, and the evolving landscape of microbiome-disease associations.

**
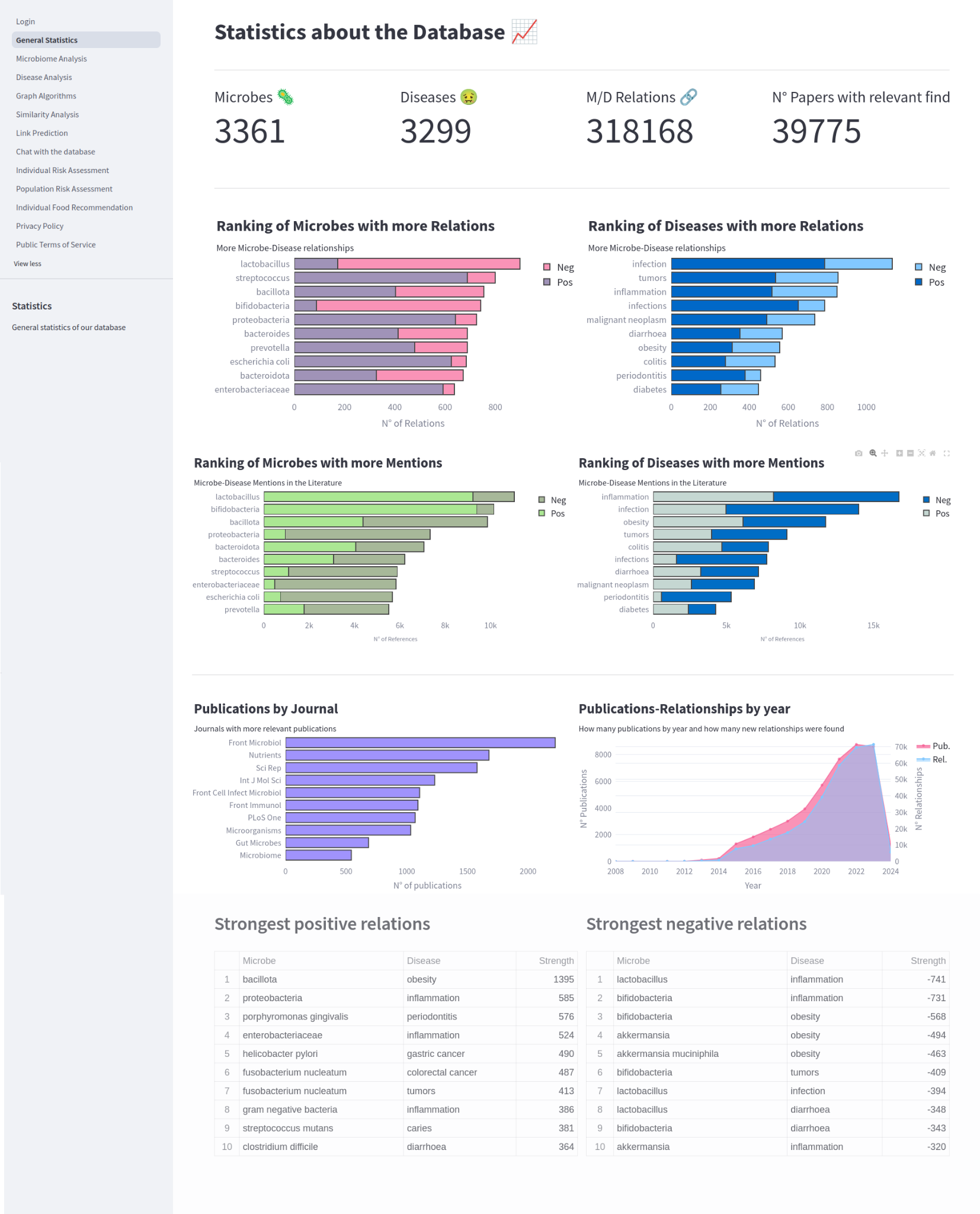
**

**Figure S3: General Statistics page**

**D.3 MICROBE- AND DISEASE-SPECIFIC ANALYSIS**

The *Microbe* and *Disease Specific Analysis* modules allow users to perform an in-depth investigation of a selected entity and its direct associations within the MINERVA knowledge graph. These tabs are designed to support both experienced researchers and users less familiar with microbiome analysis by providing an accessible yet comprehensive breakdown of relevant relationships, literature evidence, and research trends. Although Figure S4 specifically depicts the *Microbe Analysis* view, the *Disease Analysis* interface follows the same structure and functionality which is detailed in the following:

- **Part A: Basic Information ->** This section provides essential metadata about the selected entity, including its official name, concept unique identifier (CUI), and taxonomy rank (for microbes only). Definitions and recognized synonyms are also presented, which help users, especially those less familiar with taxonomic or medical terminology, clearly understand the scope and classification of the chosen microbe or disease.
- **Part B: Relationship Summary and Graph Visualization ->** Here, users are presented with two tables summarizing the most strongly supported positive and negative associations between the selected entity and other diseases or microbes. Each relationship is scored by strength, derived from the volume and consistency of literature evidence. Additionally, a graphical network visualization displays the entity at the center of a subgraph, showing its top 50 relationships, offering an intuitive understanding of its connectivity and influence across the microbiome-disease space. This dual-format presentation aids both quick exploration and detailed comparison.
- **Part C: LLM-Based Evidence Summarization ->** For frequently studied relationships with large volumes of supporting literature, this section enables users to generate a concise, evidence-based summary using an LLM given the provided API key. After selecting a microbe-disease pair, the system synthesizes multiple evidence sources to provide a readable explanation of the relationship, its biological relevance, and any conflicting findings. This feature is especially helpful for users looking to rapidly interpret complex or extensive literature without manually reviewing each paper.
- **Part D: Popularity Over Time ->** This timeline shows the number of publications per year that mention the selected entity within the context of microbe-disease relationships. By revealing trends in scientific attention, users can quickly assess whether the topic is emerging, well-established, or declining. For researchers, this can help identify hot topics or gaps in the literature; for clinicians or new users, it gives temporal context to the relevance of the entity.
- **Part E: Evidence Table ->** A detailed, filterable table listing every microbe-disease relationship involving the selected entity as extracted from the literature. Each row includes the PubMed ID, the polarity of the relationship (positive or negative), the type of source, journal name, article title, and the exact sentence from which the evidence was drawn. This transparent evidence trail supports validation and reproducibility and allows users to drill down to the source of any association.

Overall, this module enables users to perform a focused and efficient literature-driven analysis of any microbe or disease, offering not only a quantitative snapshot of relationships but also the ability to interpret them in context using modern AI tools and visualizations.

**
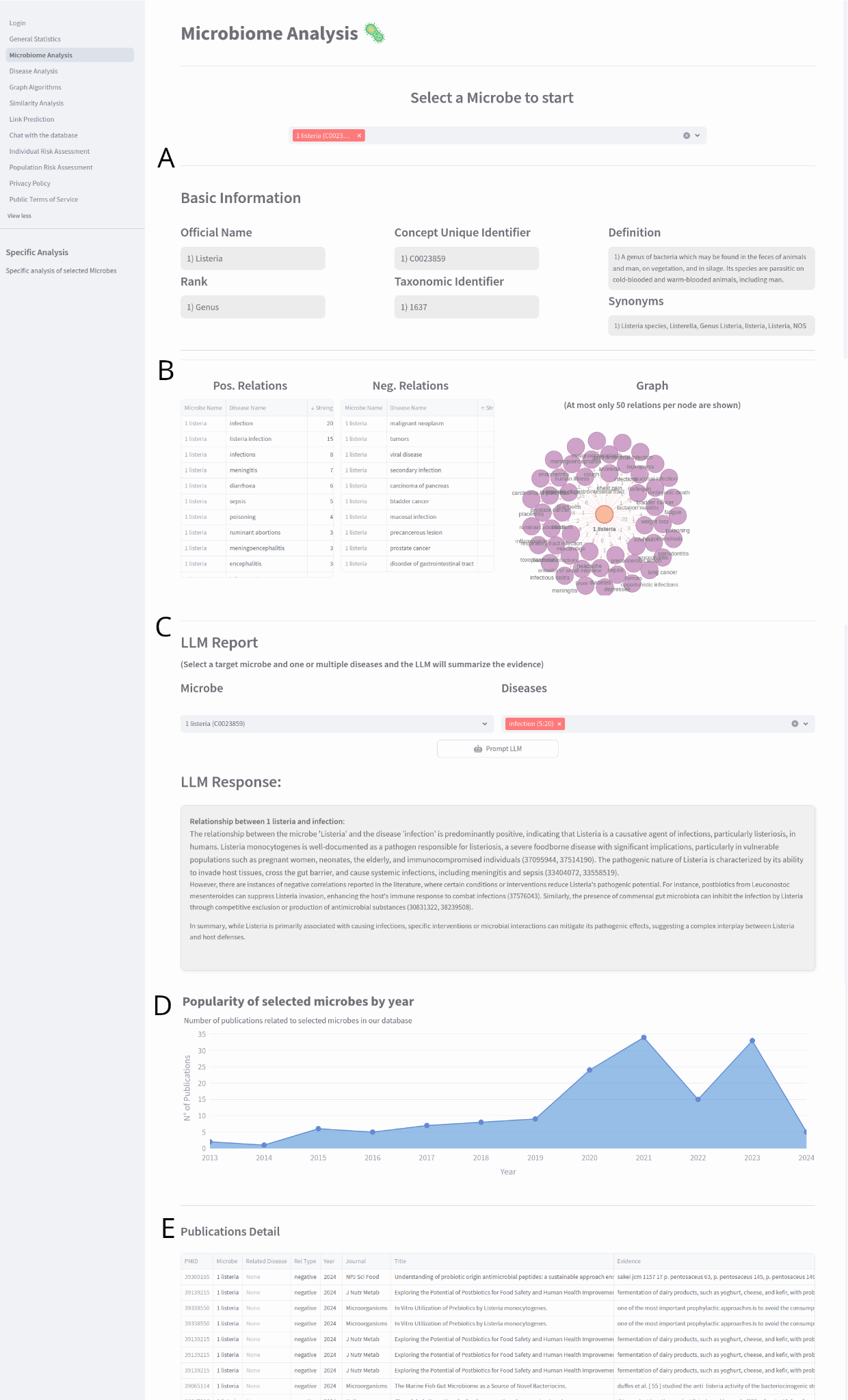
**

**Figure S4: Specific Analysis page**

**D.4 GRAPH ANALYSIS**

Figure S5 illustrates the *Graph Analysis* module, where users can explore indirect or multi-step associations between microbes and diseases using Dijkstra’s shortest path algorithm. This tool allows the identification of the shortest connection between any two entities in the knowledge graph, whether starting from a microbe and ending at a disease, or vice versa. Users first define the source and target nodes by selecting their type (microbe or disease) and specific identity using concept identifiers. Once selected, MINERVA computes the shortest path connecting the two entities and visualizes it along with other possible paths in the form of a directed network.

This module is particularly valuable for uncovering non-obvious, indirect associations that might not appear in direct microbe-disease relationships extracted from the literature. For example, identifying intermediate nodes in the graph, such as shared associated diseases, co-occurring microbes, or bridge entities, can suggest potential mechanistic pathways, comorbidities, or ecological interactions. Such insights may support hypothesis generation around second- or third-order relationships that remain underexplored or undocumented in existing studies.

**
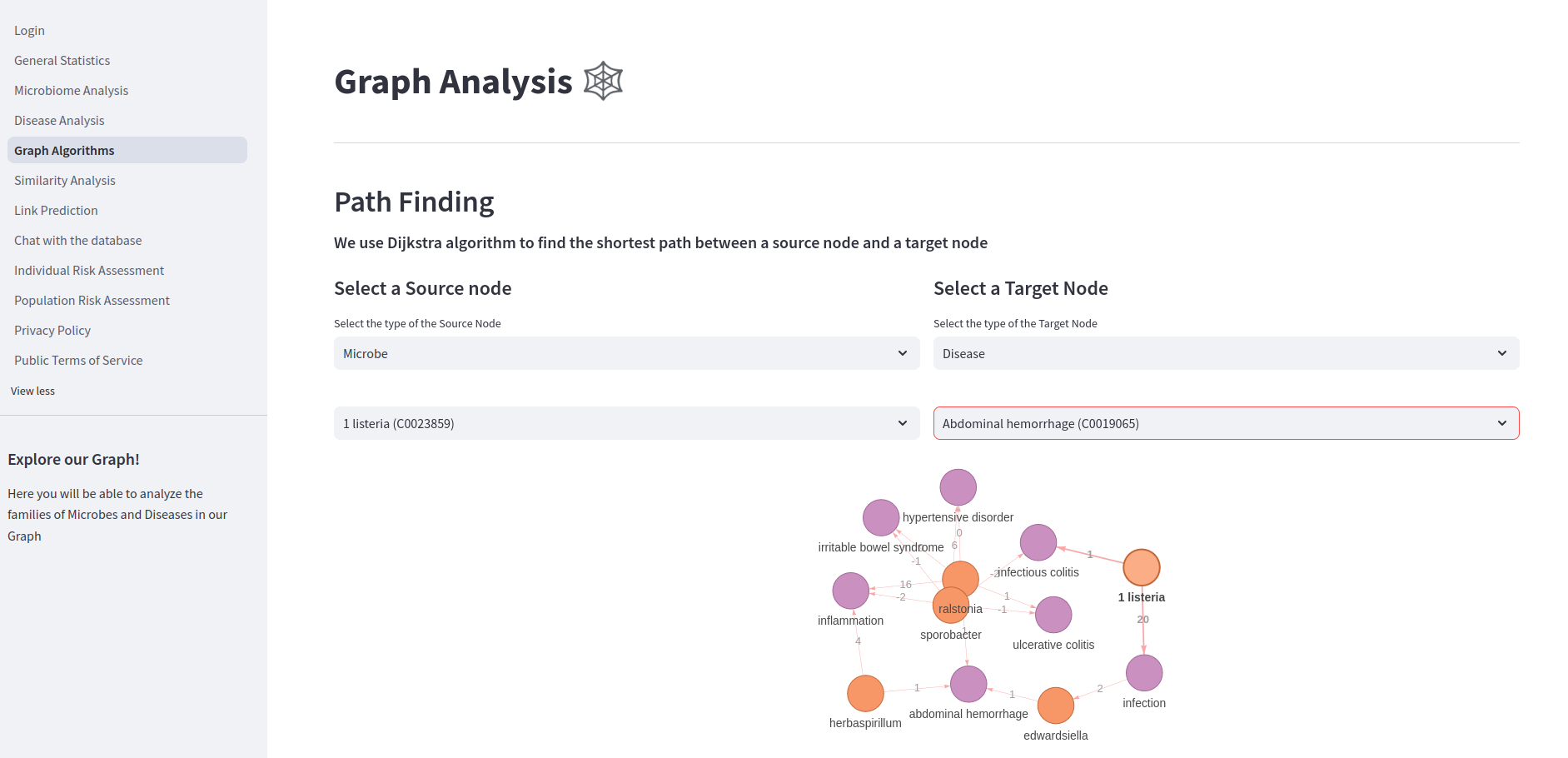
**

**Figure S5: Graph Analysis page**

**D.5 SIMILARITY ANALYSIS**

The *Similarity Analysis* module enables users to explore latent relationships among microbes or diseases by embedding the nodes of the MINERVA knowledge graph into a continuous vector space using graph embedding algorithms. As shown in Figure S6, users can select from multiple embedding methods and specify the desired number of clusters. The platform then applies unsupervised clustering to group entities based on their learned vector representations, which reflect their connectivity patterns within the graph.

This approach allows users to identify non-obvious groupings of microbes that may not be directly connected via taxonomy or known ontologies but that share similar roles in disease relationships or exhibit comparable network behavior. For example, microbes that co-occur in similar disease contexts or share overlapping comorbidity profiles may cluster together, even if they belong to distinct taxonomic branches. The interactive scatterplot (right panel) visualizes the resulting clusters in two dimensions, while the table (left panel) lists the individual entities in the selected cluster.

This functionality is particularly useful for hypothesis generation, such as identifying functionally similar microbes or diseases that could play redundant or synergistic roles, suggesting alternative biomarkers or therapeutic targets. It is also accessible for users with limited experience in graph theory, offering an intuitive interface to inspect and compare cluster composition across different embedding strategies.

**
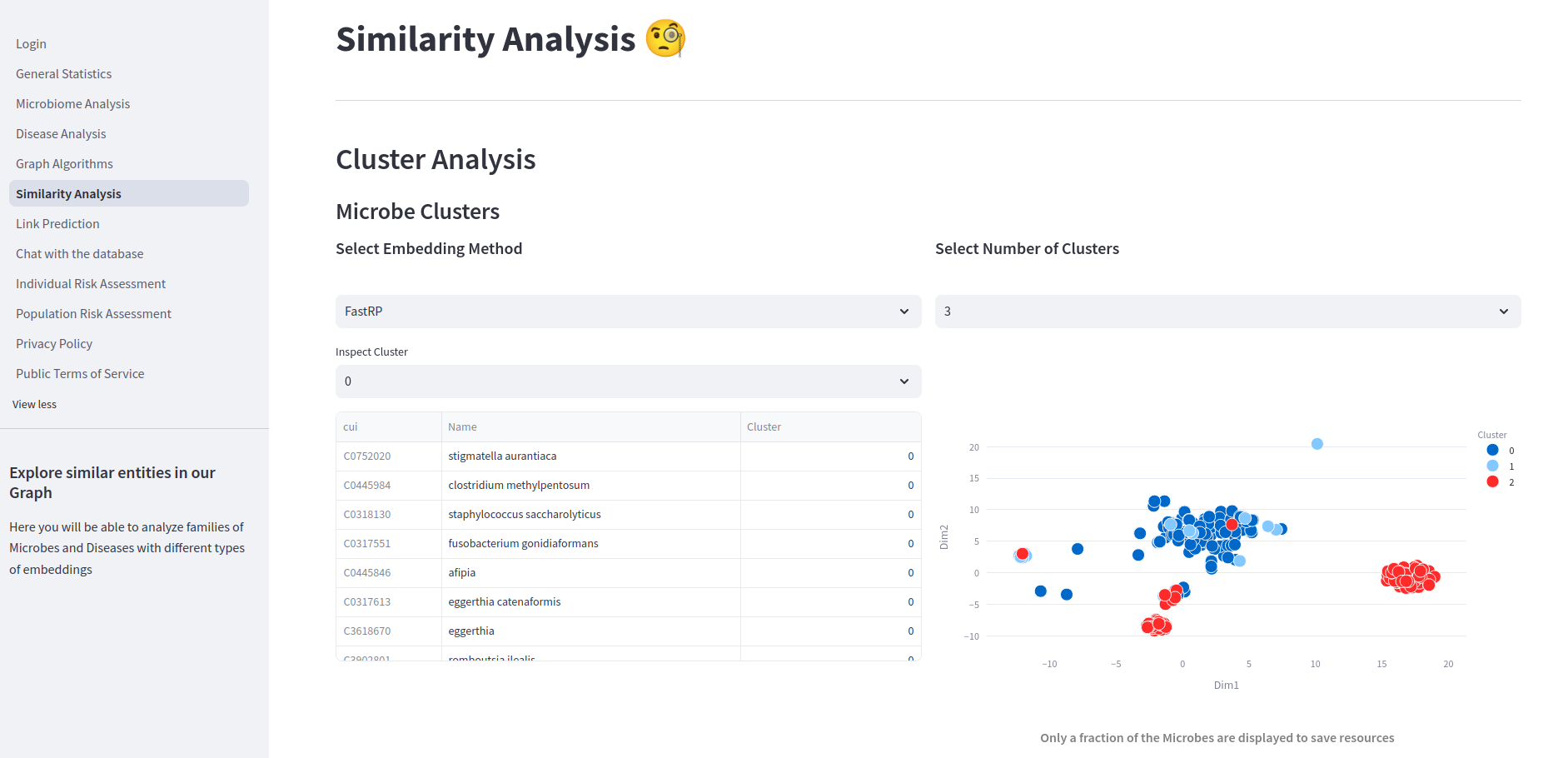
**

**Figure S6: Similarity Analysis page**

**D.6 LINK PREDICTION MODULE**

As shown in Figure S7, the Link Prediction module offers two complementary functionalities that leverage graph-based machine learning to uncover hidden patterns and suggest novel relationships within the microbiome-disease network.

- **Part A: Nearest Neighbors Analysis ->** This section uses graph embedding techniques to compute the vector similarity between a selected microbe and other microbes in the graph. Unlike the Similarity Analysis module, which focuses on cluster structure, this view ranks the most similar microbes based on their embedding proximity and reports a quantitative similarity score for each. The accompanying scatterplot helps visualize where the selected microbe lies within the global embedding space. This tool can help users identify functionally or ecologically similar microbes, which may share disease associations or occupy similar niches, even if they are not taxonomically related or directly connected in the literature. It is especially useful for hypothesis generation and comparative analysis of lesser-known taxa.
- **Part B: Predictive Link Inference ->** This section leverages a custom-trained Graph Neural Network (GNN) (detailed in Supplementary Material A) to predict potential microbe–disease associations for a selected microbe or disease. The table at the bottom of Figure S7 displays the top-ranked positive and negative predictions (i.e., potential disease links) with a model confidence above 70%. Predictions may include underexplored or novel associations not present in our knowledge base.

The *Link Prediction* functionality of MINERVA can assist researchers in prioritizing hypotheses for experimental validation, identifying novel biomarkers, or suggesting potential roles for poorly characterized microbes or diseases. It offers a powerful way to go beyond the current literature and uncover hidden or future-facing connections within the microbial knowledge graph.


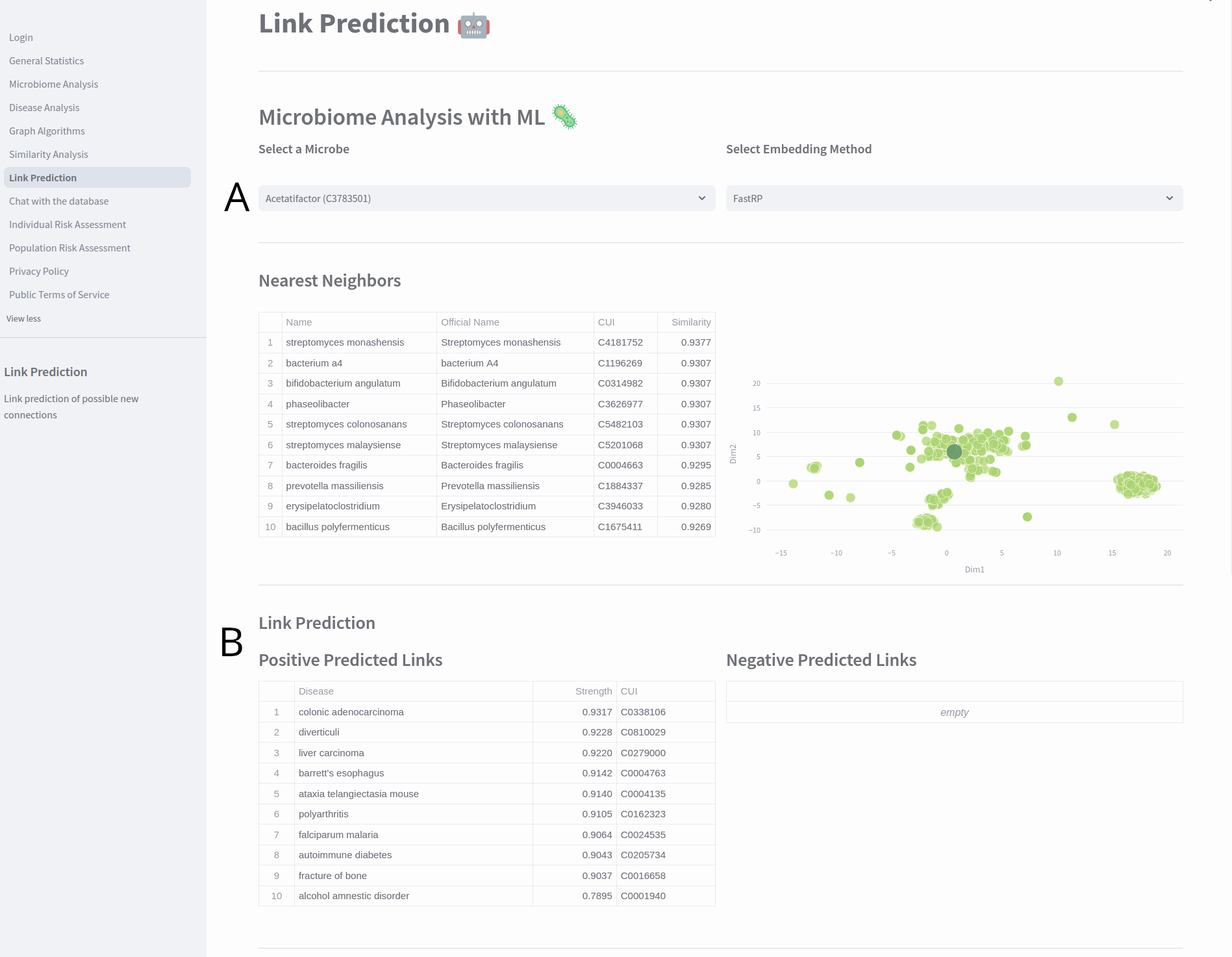


**Figure S7: Link Prediction page**

**D.7 CHAT MODULE**

The *Chat* module integrates an LLM that is tightly grounded in the MINERVA knowledge graph. Through a secure connection to the underlying Neo4j database, the LLM is able to retrieve real-time, structured information based on user queries. This enables users to easily explore microbe–disease relationships, obtain ranked associations for specific entities, and generate natural language summaries of the supporting literature.

In addition to database access, the LLM is equipped with a Wikipedia connector, which allows it to retrieve concise definitions and background information for unfamiliar biomedical terms or microbial taxa. This feature can serve to enhance the accessibility of the platform for users with less domain expertise, helping them better understand the biological or clinical significance of the entities being discussed.

As shown in Figure S8, users can interact conversationally with the system by entering free-text queries. The LLM then organizes the findings into structured outputs, highlighting positive and negative associations, strain-level variation, and context-dependent relationships (e.g., by geography or diet). Quantitative relationship strengths are also provided where available, alongside references to supporting publications. This module is particularly valuable for rapid evidence synthesis, hypothesis exploration, and decision support, especially when dealing with entities that have complex or contradictory literature.


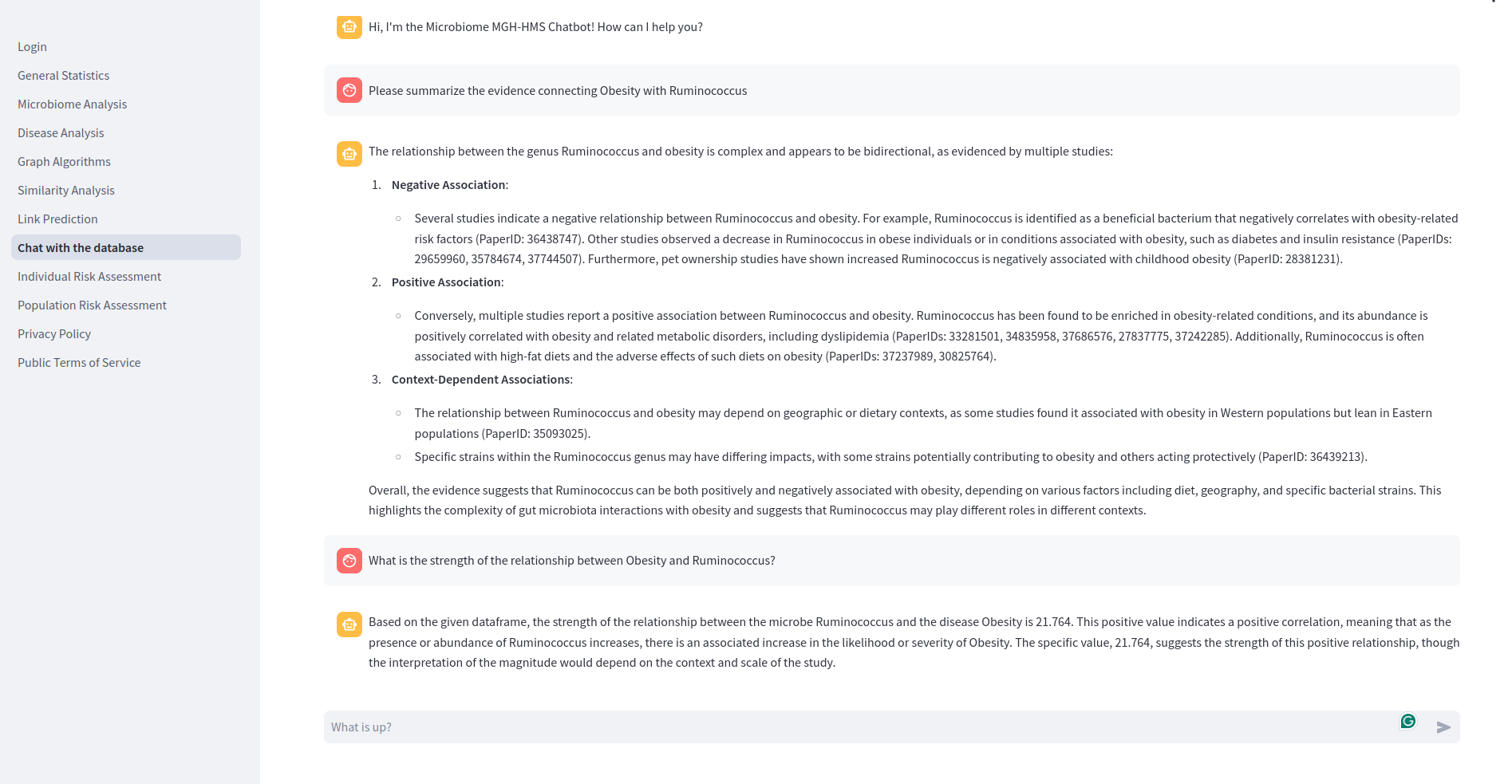


**Figure S8: Chat with the Database page**

**D.8 INDIVIDUAL RISK ASSESSMENT MODULE**

This section describes the workflow for analyzing personal microbiome compositional data using MINERVA’s Individual Risk Assessment module. We begin by outlining the data upload process and input requirements, followed by a case study involving an individual diagnosed with Alzheimer’s disease (SRR8061715) from the Data Repository for Human Gut Microbiota (Project PRJNA496408), processed through the platform.

**D.8.1 DATA UPLOAD AND PROCESSING**

MINERVA supports both personalized and population-level microbiome risk assessment through the upload of microbial composition data. The platform currently accepts relative abundance data at the genus level, which can be submitted either manually via the web interface or through a structured CSV file. Uploaded CSV files must include two columns: ncbi_taxon_id, which corresponds to the NCBI Taxonomy identifier for each genus, and relative_abundance, which should be expressed either as a percentage (e.g., 5.3) or as a decimal fraction (e.g., 0.053). Upon upload, MINERVA automatically normalizes the values so that the total abundance sums to 100% and maps each taxon to its internal knowledge base for downstream analysis.

For comparative analysis, users are required to select a reference (control) cohort against which their uploaded sample(s) will be evaluated. MINERVA offers two reference options. The first is a set of standardized healthy control groups integrated from the GMRepo database [17]. These reference cohorts consist of curated healthy individuals, and users can filter the reference population based on metadata such as sequencing type (16S rRNA or whole-genome metagenomics), sex, age, body mass index (BMI), and country of origin. This enables users to construct demographically and technically appropriate control groups tailored to their samples. The system then calculates baseline abundance distributions, including interquartile ranges (IQRs), for each genus within the selected reference group.

The second option allows users to upload a custom control group using the same file format. This is particularly useful for internal case–control studies or comparisons involving matched experimental cohorts. Whether using a GMRepo-based or user-defined reference, MINERVA compares the uploaded profile to the selected baseline and identifies genera with statistically significant deviations in abundance.

When taxa present in the uploaded target profile are absent from the selected control group, MINERVA flags these as discrepancies. These taxa are retained for certain exploratory analyses (e.g., alpha diversity or diversity-based clustering) if disease associations exist within the knowledge graph. However, taxa without supporting literature or database references are excluded from downstream disease risk prediction to maintain interpretability and avoid speculative inference

| **ncbi_taxon_id** | **relative_abundance** |
| --- | --- |
| 1678 | 12.7199 |
| 102106 | 0.314502 |
| 84111 | 0.244613 |
| 133925 | 0.885265 |
| 838 | 2.21316 |
| 1253 | 0.232964 |
| 1301 | 0.430984 |
| 189330 | 0.768783 |
| 841 | 0.675597 |
| 216851 | 2.6092 |
| 204475 | 0.966803 |
| 970 | 0.570763 |
| -1 | 136.331 |
| 816 | 0.104834 |
| 1263 | 5.75422 |
| 1485 | 3.34304 |

**Table S3: Microbiome abundance data for the Alzheimer’s disease patient (SRR8061715)**

In this example, data from an Alzheimer's disease individual (SRR8061715) obtained from the Data Repository for Human Gut Microbiota's Project PRJNA496408 is analyzed as an example of the module usage (Experiment type: Amplicon; Instrument model: Illumina MiSeq; Geolocation: China). The microbiome abundance data for this patient is shown in Table S3.

In this module, users can input their microbial composition (Figure S9). Prior to entering individual microbiome data, demographic information for the healthy reference population must be specified. Selecting *Select all* displays the microbiome distributions for the entire healthy population using boxplots.

**
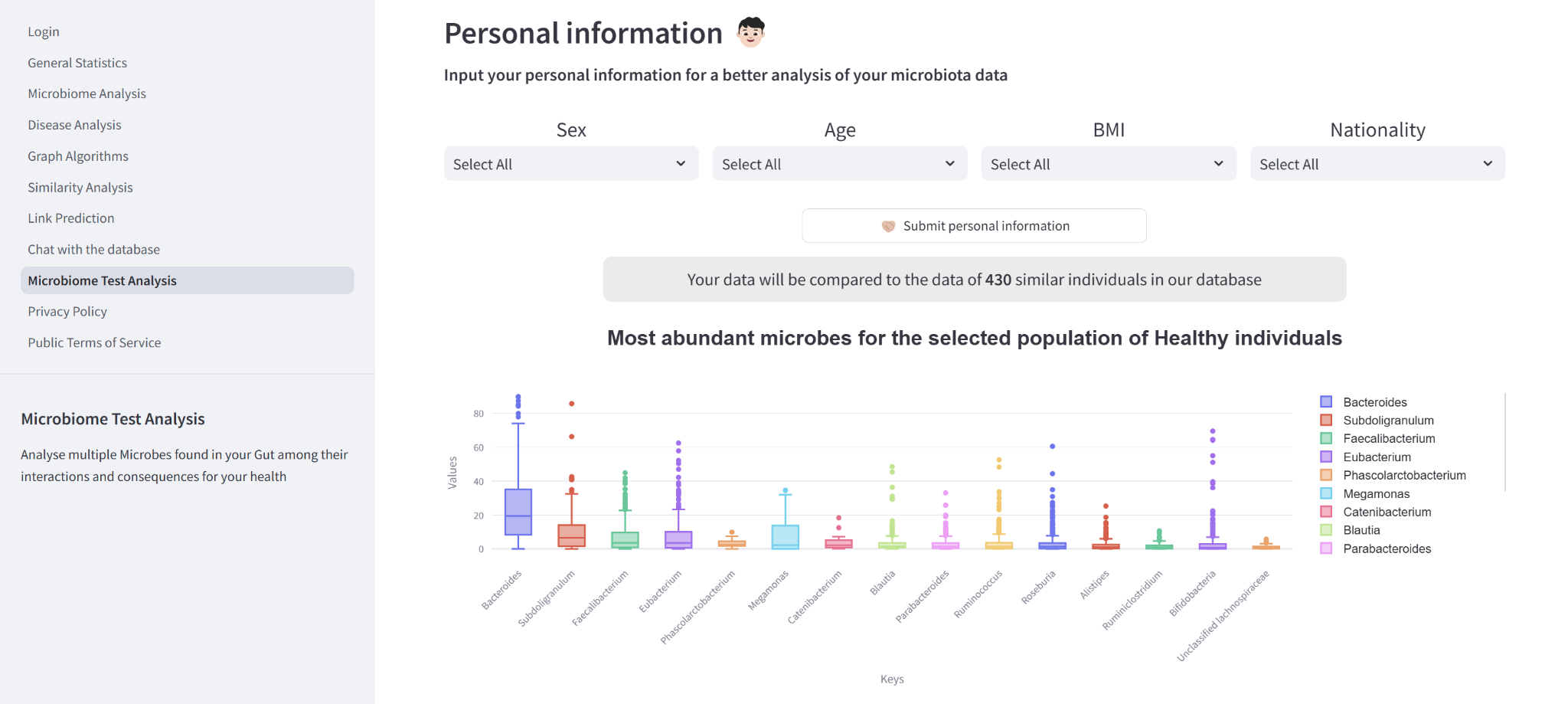
**

**Figure S9: Personal information input interface and selection of healthy reference demographics.**

Next, the *Add Microbe* button allows users to search for microbiome names and input relative abundances from personal samples. For this study, microbiome data from Table S3 was manually entered into the system. Alternatively, users can prepare a CSV file containing personal microbiome abundance data structured with two columns: *ncbi_taxon_id* and *relative_abundance*. By uploading this file to MINERVA, information can be entered easily without the need for manual input.

Upon clicking the *Analyze* button, the system shows the personal microbiome abundances relative to the interquartile range (IQR) of the healthy reference data (Figure S10). Values within the IQR are classified as normal, while those outside are flagged as too high or too low. For the Alzheimer's disease patient (SRR8061715), the abundances of Olsenella, Gemmiger, Selenomonas, and Paraclostridium exceeded the normal range, falling above the 95th percentile, whereas Bacteroides was below the normal range, falling below the 5th percentile.


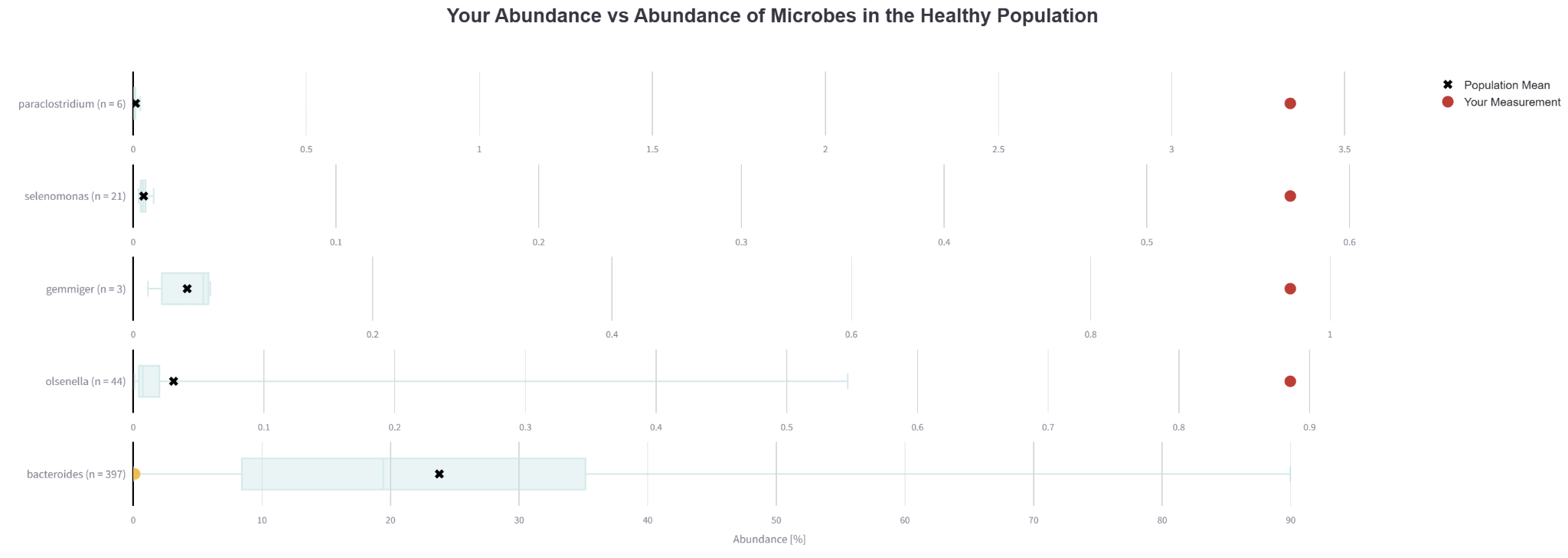


**Figure S10: Position of personal microbiome abundances relative to the healthy reference IQRs.**

The disease risks identified by MINERVA were subsequently summarized (Figure S11). A notable observation was the high risk of obesity, which aligns with previous studies [5, 6, 7]. Conversely, the patient exhibited a low risk for malignant neoplasms, consistent with reports indicating a reduced likelihood of cancer development in Alzheimer's disease patients [8, 9, 10]. Traditionally, gaining such insights from human personal microbiome abundance data would necessitate extensive literature reviews. MINERVA simplifies and accelerates this process, providing an efficient platform for in-depth analysis. Additionally, using an LLM, MINERVA generates detailed summary reports with references to further support personalized microbiome analysis (Figure S12).


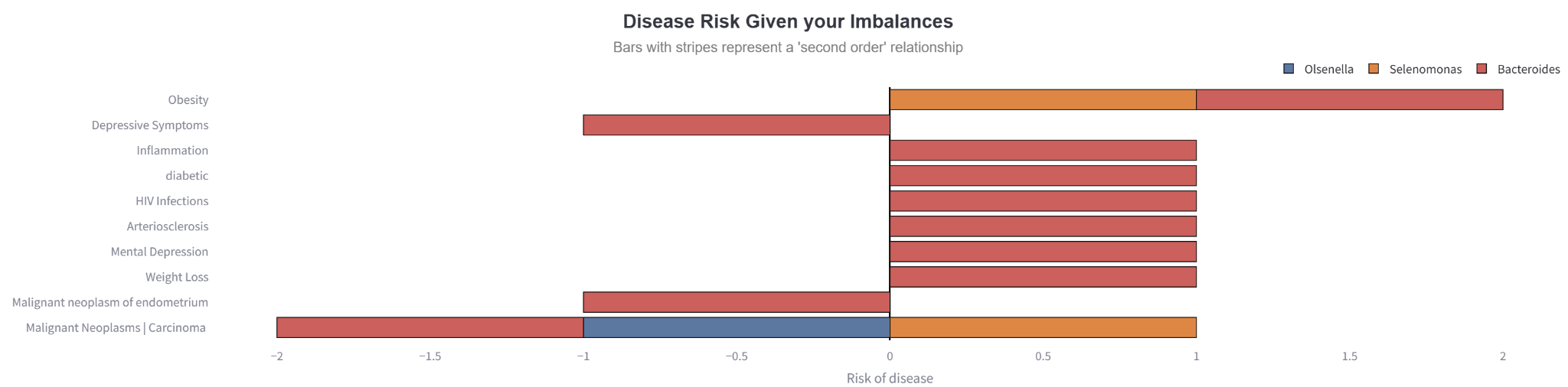


**Figure S11: Disease risk summary from MINERVA for the Alzheimer's disease patient (SRR8061715).**

**
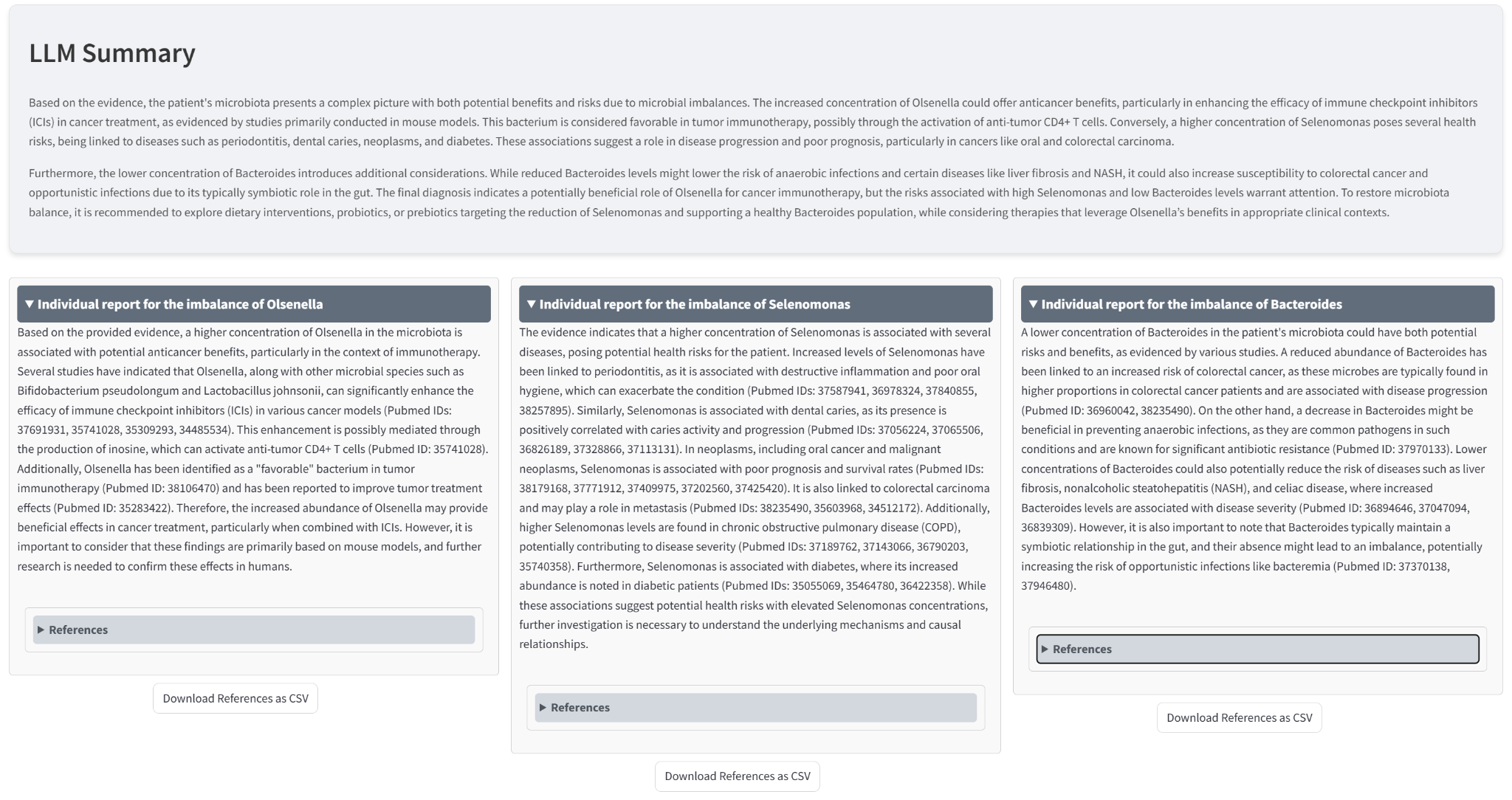
**

**Figure S12: Example of a personal microbiome analysis report generated with a large language model.**

**D.9 RESULTS FOR THE POPULATION RISK ASSESSMENT MODULE**

This study explores the outcomes of a case study utilizing the *Population Risk Assessment Module* to analyze the microbiome composition of populations. Specifically, data were drawn from the Data Repository for Human Gut Microbiota's Project PRJNA496408, which involved Amplicon sequencing performed using the Illumina MiSeq platform, with samples sourced from a geolocation in China. The dataset included 32 individuals with amnestic mild cognitive impairment (MCI) and 33 individuals diagnosed with Alzheimer’s disease (AD). It is important to note that the data and associated publication [11] used in this study were independent of the datasets utilized during the development of the MINERVA system.

The objective of the analysis was to identify disease-specific microbial imbalances (at the genus level) that could elucidate the role of microbiota in Alzheimer’s disease progression and cognitive impairment risks at a group level. This analysis provided valuable insights into the evolving microbial signatures associated with disease development.

In its current configuration, the MINERVA system allows the analysis of a single target condition at a time. Accordingly, two separate analyses were conducted: one comparing Alzheimer’s disease patients against a healthy reference population and another comparing individuals with impaired cognition to the same healthy reference population. This study presents and contrasts the insights gained from both analyses.

The Venn diagram analysis (Figure S13) reveals comparable numbers of differentially abundant microbes unique to each disease state when compared to the healthy population. Notably, the genus *Hespellia* emerged as the sole taxonomic group present in both the cognitive impairment and Alzheimer's disease cohorts while being absent in the healthy reference population. Although, to the authors' knowledge, direct associations between *Hespellia* and these specific neurological conditions have not been previously documented in the literature, although there is some evidence that suggests a potential link between this genus and another neurological condition such as Parkinson's disease [12]. The consistent presence of *Hespellia* across both cognitive impairment and Alzheimer's disease warrants further investigation to elucidate its potential role in the progression of cognitive decline and its possible mechanistic involvement in neurodegenerative processes.


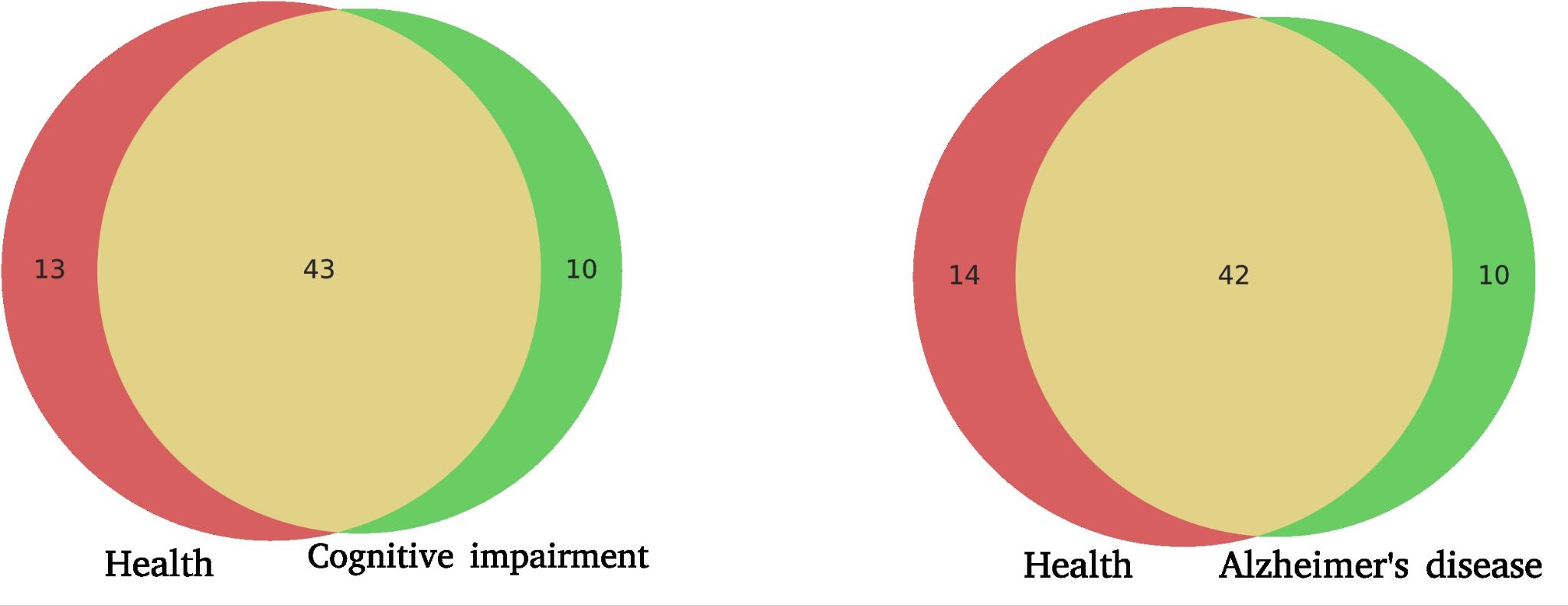


**Figure S13: Venn Diagram generated by MINERVA to compare Genus present in the target and control populations.**

Analysis of microbial diversity using MINERVA revealed both $\alpha$ and $\beta$-diversity patterns across the study populations. The $\alpha$-diversity metrics (Figure S14) showed largely comparable diversity levels between disease states and the control group, with the notable exception of Simpson's Index, which demonstrated significant differences between the Alzheimer's disease and healthy populations. Assessment of $\beta$-diversity through PERMANOVA analysis of Bray-Curtis dissimilarity matrices indicated no significant differences in community composition between the studied populations.


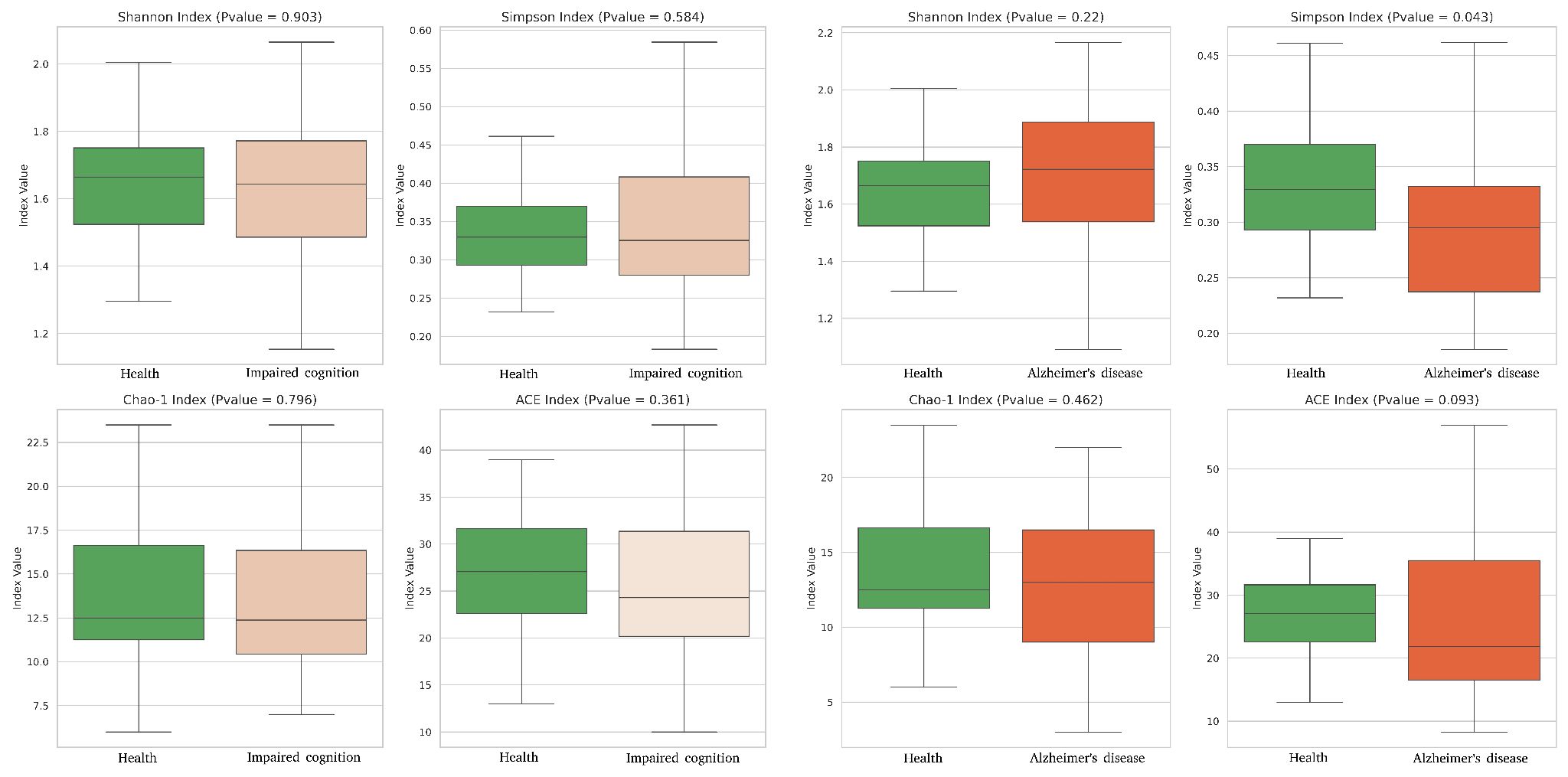


**Figure S14: Different** $\alpha$**-diversity metrics calculated by MINERVA comparing the target population with the control group.**

Group-level analysis using Variable Importance in Projection (VIP) scores derived from Partial Least Squares-Discriminant Analysis (PLS-DA) (Figure S15) revealed distinct microbial signatures for each condition. The comparison between cognitive impairment and the healthy reference population identified *Ruminococcus*, *Bacteroides*, and *Defluviitalea* as the most discriminative genera. Conversely, *Bacteroides*, *Paraprevotella*, and *Defluviitalea* emerged as the key discriminative genera between Alzheimer's disease and healthy reference populations. The condition-specific importance of *Ruminococcus* in cognitive impairment and *Paraprevotella* in Alzheimer's disease suggests these genera may serve as potential biomarkers in the progression of cognitive decline.

Now, at the individual level, Figure S16 presents the distribution of microbiome abundances associated with both conditions. Microbial signatures linked to increased risk, marked in red, were primarily driven by *Bacteroides* in both MCI and AD patients. However, differences emerged between the two groups: An abnormal abundance of *Butyricicoccus* was identified as a contributing factor to AD risk [13] in patients with MCI, while abnormal abundances of Roseburia and Ruminococcus were identified as additional contributors to AD risk [14, 15], particularly in AD patients. This shift highlights the importance of stage-specific microbial profiling in understanding and mitigating Alzheimer’s disease risk.


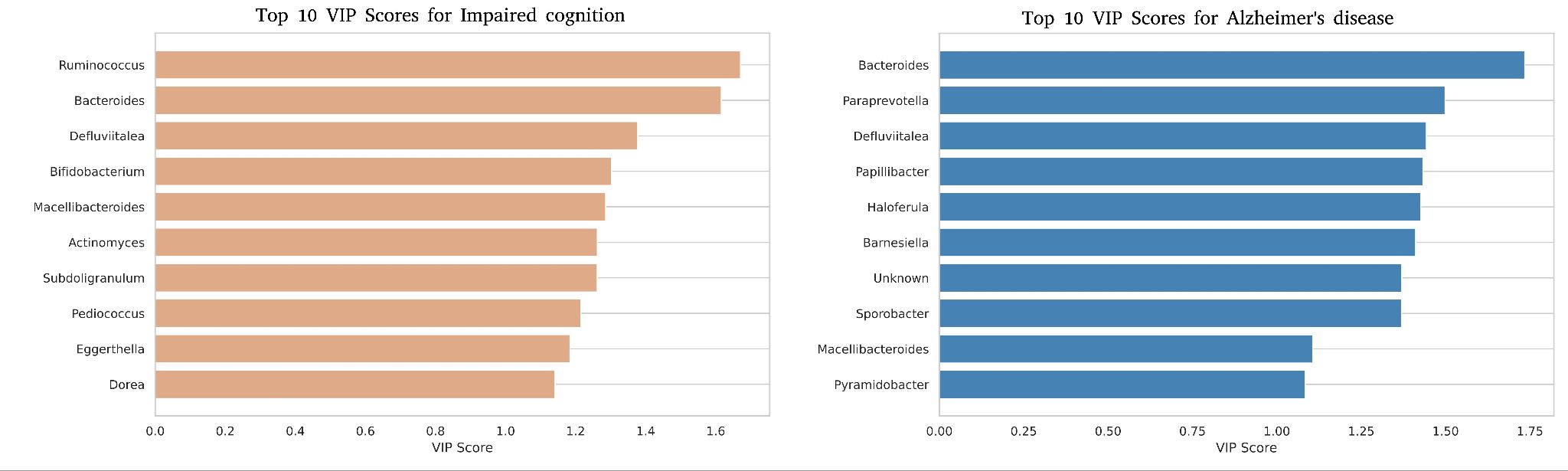


**Figure S15: Variable importance in projection scores to show the most discriminative Microbes between the target and control populations, as determined by Partial Least Squares-Discriminant Analysis (PLS-DA).**


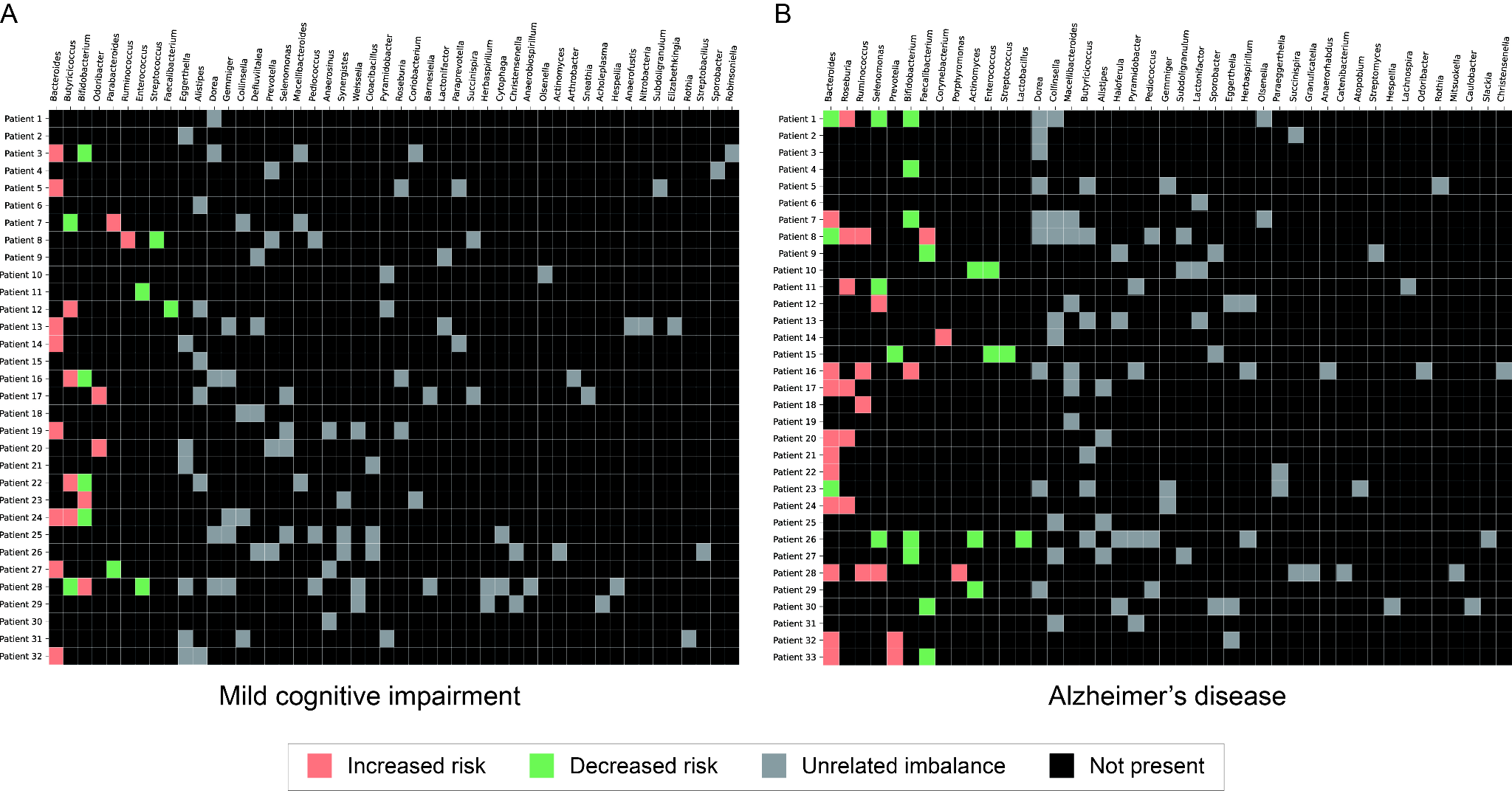


**Figure S16: Alzheimer’s disease risk induced by patient-level microbiome imbalances in MCI and AD.** Heatmaps display Alzheimer’s disease risk induced by abnormal microbiome imbalances. (A) MCI patients and (B) AD patients are shown. Red boxes represent risk-increasing microbes, green boxes indicate risk-decreasing microbes, gray boxes denote unrelated imbalances (i.e., genera with abnormal abundance with respect to controls that are not linked to Alzheimer’s or MCI in the current MINERVA knowledge base), and black areas show the absence of corresponding microbe abundance data.

The relative rankings of disease-associated risks in the two patient groups are illustrated in Figure S17. Emotional and fatigue-related conditions, such as anxiety, depressive symptoms, and fatigue, ranked highly in MCI patients, reflecting their close association with early disease stages. By contrast, systemic and metabolic conditions, including osteoporosis, weight gain, and dysbiosis, were prominent in the AD group. Notably, Alzheimer’s disease itself exhibited consistently high rankings, accompanied by a broader spread of risk-related conditions compared to MCI. This increasing heterogeneity in AD patients suggests a diversification of comorbidities as the disease progresses, pointing to the cumulative effects of long-term microbial dysregulation.

It is important to note that MINERVA’s risk assessment approach has methodological limitations that may lead to false positives. Risk predictions are based on deviations in microbial abundances relative to a healthy reference group and their known associations with diseases in the literature. This means that if a sample shows microbial patterns similar to those linked with a given disease, even if unrelated clinically, it may be flagged. For example, colorectal carcinoma appears in Figure S17, likely due to overlapping microbial signatures shared with other inflammatory or metabolic conditions.

Such predictions do not imply diagnosis, but reflect literature-based associations and potential comorbidity signals. Accordingly, these results should be interpreted as hypothesis-generating, and further validated through clinical or experimental studies before drawing strong conclusions.


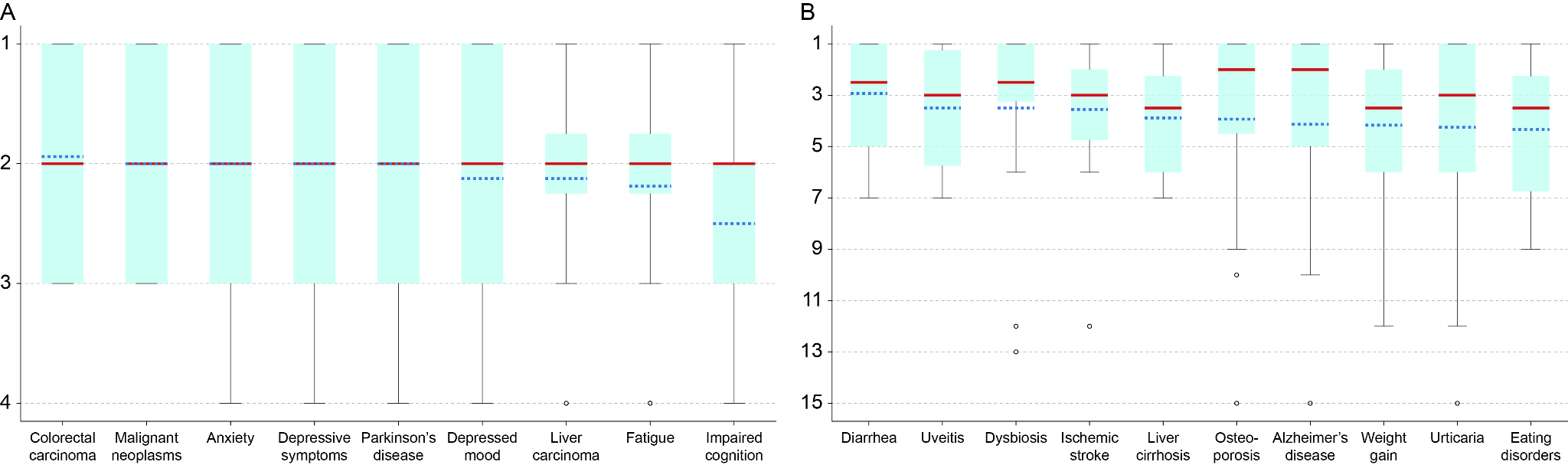


**Figure S17: Ranking of disease-associated risks in MCI and AD patients. Boxplots show the ranking of disease risks in (A) MCI and (B) AD patients. Red lines indicate medians, and blue dashed lines represent means.**

Overall, the findings from this small-scale case study demonstrate the utility of MINERVA for population microbiome analysis. Distinct microbial imbalances and their associated disease risks were observed across both group and individual levels, providing new insights into the evolving gut microbiome contributions to Alzheimer’s disease pathogenesis.

**References**

1. Fey M, Lenssen JE. Fast Graph Representation Learning with PyTorch Geometric. In: ICLR2019 Workshop on Representation Learning on Graphs and Manifolds; 2019.
2. Wang, W., Wei, F., Dong, L., Bao, H., Yang, N., & Zhou, M. (2020). MINILM: deep self-attention distillation for task-agnostic compression of pre-trained transformers. In *Proceedings of the 34th International Conference on Neural Information Processing Systems*. Curran Associates Inc..
3. Grover A, Leskovec J. node2vec: Scalable Feature Learning for Networks. In: Proceedings of the 22nd ACM SIGKDD International Conference on Knowledge Discovery and Data Mining. KDD ’16. New York, NY, USA: Association for Computing Machinery; 2016. p. 855–864.
4. Dong Y, Chawla NV, Swami A. metapath2vec: Scalable Representation Learning for Heterogeneous Networks.In: Proceedings of the 23rd ACM SIGKDD International Conference on Knowledge Discovery and Data Mining. KDD ’17. New York, NY, USA: Association for Computing Machinery; 2017. p. 135–144.
5. Terzo S, Amato A, Mul`e F. From obesity to Alzheimer’s disease through insulin resistance. J Diabetes Complications. 2021 Aug;35(11):108026.
6. Hinney A, Albayrak O, Antel J, Volckmar AL, Sims R, Chapman J, et al. Genetic variation at the CELF1 (CUGBP, elav-like family member 1 gene) locus is genome-wide associated with Alzheimer’s disease and obesity. Am J Med Genet B Neuropsychiatr Genet. 2014 May;165B(4):283-93.
7. Litwiniuk A, Bik W, Kalisz M, Baranowska-Bik A. Inflammasome NLRP3 Potentially Links Obesity-Associated Low-Grade Systemic Inflammation and Insulin Resistance with Alzheimer’s Disease. Int J Mol Sci. 20212 May;22(11).
8. Shi Hb, Tang B, Liu YW, Wang XF, Chen GJ. Alzheimer disease and cancer risk: a meta-analysis. Journal of Cancer Research and Clinical Oncology. 2015 Mar;141(3):485-94.
9. Ou SM, Lee YJ, Hu YW, Liu CJ, Chen TJ, Fuh JL, et al. Does Alzheimer’s disease protect against cancers? A nationwide population-based study. Neuroepidemiology. 2012 Oct;40(1):42-9.
10. Musicco M, Adorni F, Di Santo S, Prinelli F, Pettenati C, Caltagirone C, et al. Inverse occurrence of cancer and Alzheimer disease: a population-based incidence study. Neurology. 2013 Jul;81(4):322-8.
11. Liu P, Wu L, Peng G, Han Y, Tang R, Ge J, et al. Altered microbiomes distinguish Alzheimer’s disease from amnestic mild cognitive impairment and health in a Chinese cohort. Brain, Behavior, and Immunity. 2019;80:633-43.
12. Hey G, Nair N, Klann E, Gurrala A, Safarpour D, Mai V, et al. Therapies for Parkinson’s disease and the gut microbiome: evidence for bidirectional connection. Front Aging Neurosci. 2023 May;15:1151850.
13. Nguyen TTT, Fujimura Y, Mimura I, Fujii Y, Nguyen NL, Arakawa K, et al. Cultivable butyrate-producing bacteria of elderly Japanese diagnosed with Alzheimer’s disease. J Microbiol. 2018 Aug;56(10):760-71.
14. Li H, Cui X, Lin Y, Huang F, Tian A, Zhang R. Gut microbiota changes in patients with Alzheimer’s disease spectrum based on 16S rRNA sequencing: a systematic review and meta-analysis. Front Aging Neurosci. 2024 Aug;16:1422350.
15. Heravi FS, Naseri K, Hu H. Gut Microbiota Composition in Patients with Neurodegenerative Disorders (Parkinson’s and Alzheimer’s) and Healthy Controls: A Systematic Review. Nutrients. 2023 Oct;15(20).

1. In melanoma patients responding to iCPI more abundant species included Bifidobacterium, Collinsella, Enterococcus, Clostridiales, Rominococcus and Faecalibacterium, while low levels of Akkermansia muciniphila were observed in epithelial cancers not responding to iCPI (174). [↑](#footnote-ref-1)
2. It is known that Helicobacter pylori plays an important role in the pathogenesis of ulcer, while Firmicutes are involved in obesity (Yu et al., 2014). [↑](#footnote-ref-2)
3. For example, the colonization of intestinal commensal Bacteroides fragilis protects mice from developing experimental inflammatory bowel disease via TLR2 (Round and Mazmanian, 2010). [↑](#footnote-ref-3)
4. By using animal models of experimental colitis (20) and arthritis (21), it was shown that Gram-negative bacteria, including Escherichia, reduced inflammation, possibly through the TLR2/IL-10 axis which results in the repression of the pro-inflammatory pathway (20), whereas Gram-positive bacteria such as Lactobacillus contributed to the development of a more severe disease (21). [↑](#footnote-ref-4)
5. Prevotella falsenii, Parabacteroides distasonis and Bacteroides eggerthii enhance and Alistipes finegoldii attenuates colitis in mice [↑](#footnote-ref-5)
